# Regulation of the Actin Cytoskeleton via Rho GTPase Signalling in *Dictyostelium* and Mammalian Cells: A Parallel Slalom

**DOI:** 10.3390/cells10071592

**Published:** 2021-06-24

**Authors:** Vedrana Filić, Lucija Mijanović, Darija Putar, Antea Talajić, Helena Ćetković, Igor Weber

**Affiliations:** Division of Molecular Biology, Ruđer Bošković Institute, Bijenička 54, HR-10000 Zagreb, Croatia; lucija.mijanovic@irb.hr (L.M.); darija.putar@irb.hr (D.P.); antea.talajic@irb.hr (A.T.); helena.cetkovic@irb.hr (H.Ć.)

**Keywords:** Rho, Rac, cell migration, cell motility, convergent evolution, Amoebozoa

## Abstract

Both *Dictyostelium* amoebae and mammalian cells are endowed with an elaborate actin cytoskeleton that enables them to perform a multitude of tasks essential for survival. Although these organisms diverged more than a billion years ago, their cells share the capability of chemotactic migration, large-scale endocytosis, binary division effected by actomyosin contraction, and various types of adhesions to other cells and to the extracellular environment. The composition and dynamics of the transient actin-based structures that are engaged in these processes are also astonishingly similar in these evolutionary distant organisms. The question arises whether this remarkable resemblance in the cellular motility hardware is accompanied by a similar correspondence in matching software, the signalling networks that govern the assembly of the actin cytoskeleton. Small GTPases from the Rho family play pivotal roles in the control of the actin cytoskeleton dynamics. Indicatively, *Dictyostelium* matches mammals in the number of these proteins. We give an overview of the Rho signalling pathways that regulate the actin dynamics in *Dictyostelium* and compare them with similar signalling networks in mammals. We also provide a phylogeny of Rho GTPases in Amoebozoa, which shows a variability of the Rho inventories across different clades found also in Metazoa.

## 1. Introduction

Small GTPases from the Rho family have continued to draw the attention of students of cell motility ever since the ground-breaking discovery of their paramount importance in the regulation of the actin cytoskeleton [1,2,3]. Initially demonstrated in mammalian and yeast cells, their role as the master switches in control of actin remodelling soon turned out to be universal among eukaryotes [4]. From an evolutionary perspective, it should be advantageous to compare Rho signalling in motile mammalian cells with cells of evolutionary distant organisms that share their major traits rooted in the actin cytoskeleton, such as the crawling locomotion, internalization of the extracellular material by large-scale endocytosis, intercellular and extracellular adhesion coupled with anchorage to their surroundings, and binary division by actomyosin-based constriction. Organisms belonging to nonflagellated amoebozoans fulfil these criteria rather well. Amoebozoa constitute a monophyletic clade that branched from Opisthokonta early in the evolution of eukaryotes, probably already in the Mesoproterozoic [5,6]. The lifestyle of these highly motile amoebae, e.g., of their best studied representative *Dictyostelium discoideum*, is remarkably reminiscent of the mammalian white blood cells, particularly neutrophils and phagocytes [7]. Similar to the cells that perform immune surveillance in complex multicellular organisms, *Dictyostelium* amoebae chase their microbial prey by chemotaxis and engulf it by phagocytosis [8,9]. During their starvation-induced aggregation, the amoebae adhere to each other and move in multicellular streams reminiscent of collective cell movements during animal embryogenesis [10,11,12]. The amoeboid locomotion of *Dictyostelium* cells and their capability to internalize nutrients by macropinocytosis very closely match the corresponding acquired traits of cancer cells [13,14,15,16,17,18] and, more generally, mirror the mesenchymal-amoeboid transition occurring during embryonal development [13,19,20].

The shape and locomotion of *Dictyostelium* amoebae are astonishingly plastic. Although they predominantly move by protrusion of irregular pseudopodia similar to leukocytes, vegetative amoebae occasionally extend thin lamellipodia akin to fibroblasts in culture and even can assume a fan-like shape and migrate in a way that highly resembles the locomotion of skin keratocytes [21]. *Dictyostelium* cells also frequently extend numerous filopodia and under certain circumstances switch to a blebbing type of locomotion [22,23,24]. During aggregation, the amoebae move by protruding long cylindrical pseudopodia, a behaviour that augments their sensitivity to chemoattractant gradients [25]. Vegetative cells feed by large-scale endocytosis and are capable of generating phagocytic and macropinocytotic cups of various shapes and sizes, and at a high rate [26,27]. Since all these diverse protrusions are generated and supported by specifically orchestrated actin assemblies, it is not surprising that the *Dictyostelium* proteome includes representatives of major classes of actin polymerases, actin-binding proteins, and myosin motor proteins, which are the convergence points of signalling pathways that regulate the actin-based supramolecular structures [28,29].

Coincidentally, the genomes of *Dictyostelium discoideum* and *Homo sapiens* both encode 20 Rho GTPases, although the two species diverged more than a billion years ago [5,6]. Given the similarities in the cellular and cytoskeletal dynamics between the two organisms, which are known to be governed by Rho GTPases, the parallel diversification of this protein family in the two lineages probably represents a prime example of convergent evolution [30,31]. Rho family genes occur in all examined eukaryotic supergroups, leading to the conclusion that Rho GTPases were already present in the last eukaryotic common ancestor (LECA). Phylogenetic analysis distributes the sequences of genes encoding 20 human Rho GTPases between the two stem groups: the Rac group includes Rac1/2/3/RhoG, Cdc42/RhoJ/Q, RhoU/V, RhoH, and RhoBTB1/2, whereas the Rho group includes RhoA/B/C, RhoD/F, and Rnd1/2/3 [4,32]. Since Rho GTPases in non-Opisthokont lineages are more closely related to Rac proteins, it is generally accepted that Rac is the founding member of the whole family. Indeed, it is usually argued that both Rho sensu stricto (s.s.) and Cdc42 are represented only in Opisthokonta [33]. The Rho family expanded rapidly in the Metazoa around 700 million years ago, probably as a result of multiple gene duplication and lateral gene transfer events [4,34]. For example, the time of emergence of Rnd and RhoU/V subgroups is consistent with their roles in the acquisition of muscle and nerve cells, while the Cdc42 isoforms, RhoJ/Q and RhoD/F, probably emerged at the time of origin of the vertebrate central nervous system [32]. 

**Figure 1:**
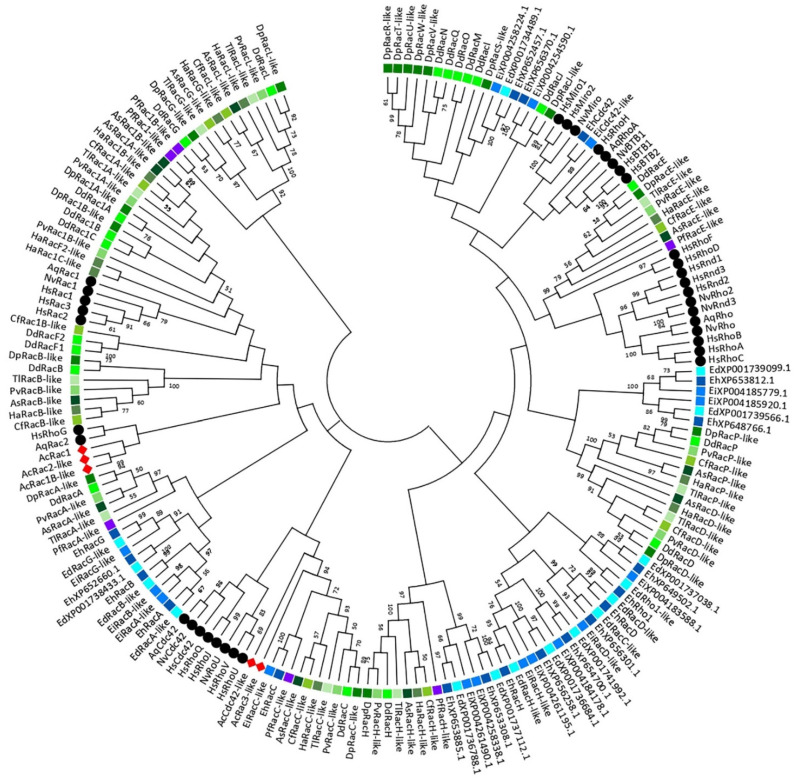
Phylogenetic tree of Rho GTPases in Amoebozoa. The evolutionary history was inferred using the maximum likelihood method and the Le_Gascuel_2008 model [36]. Evolutionary analyses were conducted in MEGAX [37], based on 186 amino acid sequences (accession numbers and sequences are presented in Supplementary File 2). Numbers at nodes are bootstrap percentages evaluated from 1000 bootstrapping replications (bootstrap values higher than 50 are indicated at the branching points). Rho GTPases from Metazoa are indicated by black circles. Amoebozoan Rho GTPases are marked as follows: subphylum Lobosa, class Discosea by red rhombus; subphylum Conosa, classes Archamoebea, Dictyostelia, and Variosea by blue, green, and purple squares, respectively.

Traditionally, Rho GTPases from *D. discoideum* were described either as Rac-like (6 members of the Rac stem group) or as RhoBTB-like (RacA), while others remained unclassified [35]. Here, we performed a phylogenetic analysis of Rho GTPases from representatives of four amoebozoan classes, with two basal metazoans and human included in the reference set (Figure 1; Table S1). Among the analysed amoebozoan genomes, 19 Rho GTPase genes were identified in the genome of *Entamoeba histolytica*, 5 in *Acanthamoeba castellanii*, and 6 in *Planoprotostelium aurantium*. This large variability in the number of Rho GTPases in amoebozoans is a sign of elevated evolutionary dynamics, similar to the high incidence of gain and loss of the Rho family members in different animal lineages [4,33]. We present evidence based on the published data that RacE and RacC GTPases from Amoebozoa, in particular from *D. discoideum*, represent bona fide functional equivalents of mammalian Rho and Cdc42 groups, respectively. It remains an open question, however, whether the Rac/Rho/Cdc42 trinity has been conserved from the common ancestor of Amoebozoa and Metazoa, or if it emerged independently in these two clades.

It is remarkable that the cells of organisms so evolutionarily distant as cellular slime moulds and mammals share so many similar traits based on a highly dynamic actin cytoskeleton. This apparent conservation of the actin-dependent cellular processes poses the question of the conservation of upstream signalling mechanisms. In this review, we systematically present the known signalling pathways in *Dictyostelium* that start with the activation of specific Rho GTPases and finish with a particular actin cytoskeleton activity, while drawing parallels with the comparable signalling networks in mammals. We arrive at a complex overall picture: while some *D. discoideum* Rho GTPases appear to be partial functional equivalents of their mammalian counterparts and engage in related signalling pathways, there is no strict one-to-one correspondence between the two, as epitomized by several original solutions on how to convey signals triggered by amoebozoan Rho GTPases to induce rearrangements of the actin cytoskeleton. Moreover, the overall picture is sparse since the mechanistic modes of action of many *Dictyostelium* Rho GTPases are still not known in any detail, and their further investigation should therefore be encouraged.

## 2. Rho GTPase Family in *Dictyostelium discoideum*

Rho GTPases constitute one of the five families within the Ras superfamily of small monomeric G proteins [38]. Ras proteins are commonly described as molecular switches, because they are active and transduce signals in the GTP-bound state [39]. They have high binding affinities for both GTP and GDP and most of them exert low intrinsic GTPase and nucleotide exchange activities. These conserved biochemical features are determined by the shared structural design of the guanine nucleotide-binding domain composed of five G-boxes [40,41]. Most Ras GTPases interact with their downstream target proteins, the so-called effectors, only in their active, GTP-bound state. Conversions between the inactive GDP-bound and the active GTP-bound conformations are catalysed by guanine nucleotide exchange factors (GEFs) that, under physiological conditions, promote GTPase activation and by GTPase activating proteins (GAPs) that promote inactivation by facilitating hydrolysis of the bound GTP [42]. Conformational differences between GDP- and GTP-bound Ras proteins are mostly confined to the two surface regions designated as switch I and switch II [39,43]. Switch regions coincide with the effector-binding domain that becomes exposed in the GTP-bound conformation.

Of note, half of the mammalian Rho family members do not conform to the classical regulation by GEFs and GAPs. These, so-called atypical Rho GTPases are predominantly present in the GTP-bound state, either because of a defective GTPase activity or due to an increased intrinsic GDP/GTP exchange activity, and are regulated by different means [44]. Rho GTPases are characterized by a 10–15 amino acid long insert that is not found in other Ras superfamily GTPases [45,46,47]. In addition, the activity of some Rho family members is further regulated by a third class of proteins, the Rho GDP-dissociation inhibitors (RhoGDIs) [48,49]. Rho effectors use various structural motifs to interact with Rho GTPases [50]. For example, some Rho effectors bind Rho GTPases via a leucine-zipper-like HR1 motif inside the Rho effector homology (REM) region, or via a ROK-kinectin homology (RKH) Rho binding domain, while many Cdc42 and Rac1 effectors bind to the cognate GTPases via a CRIB (Cdc42/Rac interactive binding) motif harboured within their GTPase binding domains (GBD) [51,52]. The GTPase–effector interaction often induces conformational changes in the effector that relieve its intramolecular autoinhibition [51]. For a comprehensive overview of the physiological functions of mammalian Rho proteins, the reader is referred to several reviews, some with a specific emphasis on the role of Rho GTPases in the regulation of the actin cytoskeleton [44,53,54,55,56].

*D. discoideum* genome contains 20 *rac* genes (*rac1A*, *rac1B*, *rac1C*, *racA*, *racB*, *racC*, *racD*, *racE*, *racF1*, *racF2*, *racG*, *racH*, *racI*, *racJ*, *racL*, *racM*, *racN*, *racO*, *racP*, and *racQ*) and 1 pseudogene (*racK_ps*) (http://dictybase.org, accessed on 4 February 2021; [57]). Their protein products are usually divided into the Rac-like subfamily (Rac1A/1B/1C, RacB, RacF1, and RacF2), the RhoBTB-like RacA, and others, allegedly with no obvious orthologues of Cdc42 and Rho [58,59]. In the following, we provide an original phylogenetic analysis of a subset of amoebozoan Rho family members (Section 3) and a succinct overview of the pathways that transduce signals from small Rho GTPases to the actin cytoskeleton in D. discoideum (Section 4), drawing parallels, when possible, with the corresponding signalling routes and functional outcomes in mammalian cells. The presented data indicate that Rho and Cdc42 bona fide functional equivalents are present in *D. discoideum* and probably also other related amoebozoan species.

## 3. A Phylogeny of the Rho GTPase Family in Amoebozoa

Although basic phylogenies of the Rho family GTPases in *Dictyostelium discoideum* and *Entamoeba histolytica* have been published [35,58,60,61], to the best of our knowledge no such analysis has been attempted at the level of Amoebozoa as a clade, i.e., by taking into account multiple species from different amoebozoan classes. Our comparative analysis of the Rho GTPase-mediated regulation of the actin cytoskeleton and cell motility in *D. discoideum* and mammalian cells (Section 4) pointed out complex functional analogies between the two systems that prompted us to construct a phylogenetic tree comprising Rho GTPases from 12 amoebozoan species belonging to two subphyla and five classes, in addition to three representative metazoans (Figure 1; Table S1). The tree and the supplementary multiple sequence alignments shed light on several hitherto neglected aspects of the variability of the Rho GTPase repertoire in Amoebozoa and exposed examples of potentially misleading misnomers in the currently utilized nomenclature.

A total set of 151 amino acid sequences from 12 different species of amoeboid protists were identified as corresponding to Rho GTPase homologs at the National Center for Biotechnology Information database (NCBI) using the *blastp* algorithm (https://blast.ncbi.nlm.nih.gov/Blast.cgi, accessed on 11 February 2021) (Table S1). In order to visualize phylogenetic relationships of the Rho homologs among different species of Amoebozoa, a phylogenetic tree was constructed using the maximum likelihood algorithm in the MEGAX software (Figure 1; [37]). For this purpose, amino acid sequences were aligned using the multiple sequence alignment with high accuracy and high throughput algorithm MUSCLE [62] against the known Rho GTPase proteins from basal Metazoa (*Nematostella vectensis* and *Amphimedon queenslandica*) [34] and a representative of higher animals, *Homo sapiens*, which served as a reference. A multiple sequence alignment of Rho family GTPases was used to identify conserved domains, indicating that all members of the Rho, Rac, Cdc42, and RhoBTB subfamilies in Amoebozoa have domains responsible for nucleotide binding and hydrolysis (G1-5 boxes) together with both switch I and II regions that specifically bind regulators or effectors. Rho insert, the most characteristic Rho family signature, is present in all analysed amoebozoan proteins, although in some cases this insert is shorter than the typical 13 amino acids. Interestingly, an alignment of RacA proteins from Dictyostelia and Variosea against human RhoBTB GTPases verified the presence of two BTB domains and their affiliation to the RhoBTB subfamily (Figure S1). Since members of the RhoBTB subfamily were not identified in the genomes of basal metazoan such as sponges, placozoans, and ctenophores [34], their presence in Amoebozoa, a clade that predates Opisthokonta, supports the suggestion that RhoBTB is one of the eldest Rac paralogs [32].

Most amoebozoan species included in our analysis belong to the subphylum Conosa and only one to the subphylum Lobosa, class Discosea [6,63,64]. Within the Conosa subphylum, our phylogenetic analysis demonstrates a divergent evolution between Rho GTPases from Archamoebea (i.e., *Entamoeba*) and other classes, namely Dictyostelia and Variosea (Figure 1). Based on a proteomic analysis, Song et al. (2005) also reported a significant divergence between *E. histolytica* and *D. discoideum* that is even deeper than between animals and fungi, although they each represent one Amoebozoa subphylum. However, the evolutionary analysis shows that Dictyostelia and Variosea Rho GTPases are most closely related and grouped together, while proteins from Archamoeba are placed at distant positions forming a few relatively independent branches. According to the phylogenetic clusters and a varying number of homologs in Amoebozoa, this is probably a consequence of lineage-specific duplications that likely happened shortly after the Amoebozoa split from the Opisthokont lineage [65]. *Acanthamoeba castellanii*, a representative of the Lobosa lineage, has the smallest set of Rho GTPases comprising only Rac and Cdc42 subfamily members, which are not grouped into clearly defined common branches with other Amoebozoa.

The nomenclature of Rho GTPases in Amoebozoa was introduced independently for each organism, mainly in original publications where the corresponding genes were initially identified and was essentially retained by convention, resulting in several misleading labels. For example, *E. histolytica* XP_654488 was labelled as EhRho1, although it harbours critical mutations in the switch I and G5 box compared to archetypical Rho proteins, and even lacks the Rho insert, a hallmark of the entire Rho family (Figure S2; [66]). It was therefore even suggested that it should be reclassified to the Ras family, but structural and functional characterization has subsequently corroborated that its initial categorization as an *E. histolytica* Rho orthologue was warranted [67]. On the other hand, *D. discoideum* XP_640935.1 contains the Rho insert and conforms to the consensus primary structure of G1–G5 and both switch regions of mammalian Rhos (Figure S2), but was nevertheless designated as RacE, and its relatedness to mammalian Rhos s.s. was, until recently, ignored, probably due to two sizeable inserts at the C- and N-termini that biased the global sequence similarity scores [68]. Whereas RacE is a clear amoebozoan counterpart of RhoA with several common functional roles, we note that amoebozoan RacC proteins might have a similar position in relation to mammalian Cdc42, although their functional similarity is less well supported by the available data (Figure S3; [69]).

In general, obtaining a clear insight into the evolutionary relationships between the analysed Rho GTPases from Amoebozoa and selected Metazoa (Porifera, Cnidaria and Mammalia) is quite daunting based on the available data, mainly due to their ancient divergence point and specific selective constraints in each of the taxonomic groups that shaped their respective Rho GTPase signalling pathways. These obstacles are reflected in low support values for most of the deeper nodes of the constructed evolutionary tree (Figure 1), indicating that the core of the tree topology is not very robust, and precluding any definitive statements regarding possible orthological relationships between the amoebozoan and mammalian Rho family GTPases: for instance, for RacE vs. RhoA and RacC vs. Cdc42. Interestingly, this statement also applies to the branches and nodes that link Dictyostelium Rac1A/B/C to human Rac1/2/3, which are short and poorly supported, indicating weak phylogenetic signals in favour of the specific orthology of the two groups. To obtain a better resolution of the evolutionary history of Rho GTPases in Amoebozoa, a more comprehensive phylogenetic analysis is required, covering the range of homologous proteins from other intermediately related organisms. Moreover, additional phylogenetic probabilistic algorithms should be employed in order to obtain robustness and support for the nodes [70]. It is also possible to remove uninformative sites from the multiple sequence alignments to get a more precise output [71].

## 4. Comparative Analysis of the Rho Signalling in *Dictyostelium* and Mammalian Cells

### 4.1. Rac1 GTPases

The three *D. discoideum* Rac1 GTPases are more than 90% identical in the amino acid sequence shared among themselves. After cloning in 1993, it became common to regard the three Rac1 isoforms as *Dictyostelium* orthologues of the human Rac1 GTPase, with whom they share around 80% amino acid sequence identity; hence, they were named Rac1A, Rac1B, and Rac1C [72]. Their expression shows an increase during multicellular development, with Rac1A being expressed much stronger than Rac1B and Rac1C, in both vegetative and developing cells [72,73,74]. Rac1B and Rac1C are induced during the sexual maturation of cells as well [75]. Early studies on cells overexpressing wild-type forms of *D. discoideum* Rac1 GTPases have demonstrated their involvement in the formation of filopodia, lamellipodia, and membrane ruffles and suggested their roles in the regulation of growth, motility, large-scale endocytosis, and development [76,77]. However, the deletion of individual *rac1* genes did not induce any prominent phenotypic changes when knockout cells were grown in suspension or on bacterial lawns, suggesting their redundancy [77,78]. The most noticeable effects were slightly smaller plaques of *rac1B*- and *rac1C*-null cells and an apparent defect in early development of *rac1B*-null cells; however, these phenotypes have not been further investigated [78]. On the other hand, biochemical studies have identified several interactors, the so-called effectors, through which Rac1 GTPases exert their functions and affect the actin cytoskeleton.

**Figure 2:**
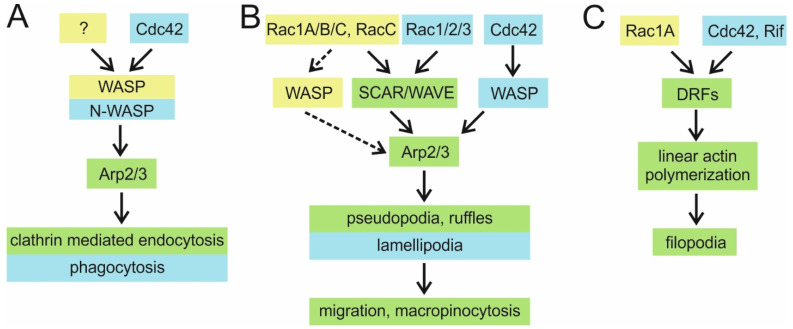
Parallel presentation of major signalling pathways starting from Rac1A/B/C and RacC in Dictyostelium (yellow) and Rac1/2/3 and Cdc42 in mammals (blue) and converging onto the actin cytoskeleton regulation. (**A**,**B**) Activation of the Arp2/3 complex via WASP family proteins, converging on the clathrin-mediated endocytosis and phagocytosis (**A**), or the protrusion of pseudopodia and macropinocytotic cups (**B**). (**C**) Activation of the DRF-mediated actin polymerization and protrusion of filopodia. The common elements shared by Dictyostelium and mammals are shown in green. The default active state of small GTPases is GTP-loaded.

It has been recognized early on that a core group of Rac/Cdc42 effectors is conserved across eukaryotes, in particular fungi and Metazoa [79]. Most of the core effectors involved in the regulation of the actin cytoskeleton are also conserved in *Dictyostelium* and will be dealt with at length in the remainder of this review. Indicatively, several core effectors interact with GTPases from both Rac and Cdc42 groups in the same organism, while other effectors interact with Rac in some organisms and with Cdc42 in others [79]. We encounter a similar situation in *Dictyostelium* (Figure 2), where it is also not possible to draw a sharp dividing line between the pathways driven by individual GTPases, neither in interaction nor in functional assays. The yeast-two-hybrid (Y2H) and pull-down assays that use CRIB motifs from different effectors as baits are especially prone to show interactions with multiple GTPases, and vice versa [59,80,81,82,83,84,85]. Refining the existing map of functional interactions between individual GTPases and their effectors in *Dictyostelium* therefore remains an important task for future research.

#### 4.1.1. WASP Family Proteins as Rac1 Effectors

A group of the most prominent effectors of mammalian Rac1 and Cdc42 are the actin filament nucleation promoting factors (NPFs) of the WASP family. NPFs stimulate the nucleation activity of the intrinsically almost inactive Arp2/3 complex that nucleates the branching actin filament on the sides of existing mother filaments [86,87]. WASP protein family in mammals encompasses at least six members that often have multiple isoforms: WASP, N-WASP, WAVE/SCAR, WASH, and metazoa-specific JMY and WHAMM, where WASPs and WAVEs are regulated by Rho GTPases. They all have a carboxyl terminal WCA domain that potently activates the Arp2/3 complex and a central PRD domain, whereas their amino termini differ, reflecting different modes of regulation and distinct means of spatiotemporal activation [88]. In general, in addition to Rho GTPases, various kinases and phosphoinositides regulate the activity of NPFs. In their unstimulated state, WASP and N-WASP are autoinhibited by an intramolecular interaction between the WCA and GBD domains, which precludes their interaction with the Arp2/3 complex [89,90,91]. The binding of activated Cdc42 or Rac1 relieves this autoinhibition and, in synergy with additional signals, stabilizes the active conformations of WASP and N-WASP [88,91,92,93].

Human and *Dictyostelium* WAVE proteins were cloned almost at the same time [94,95]. In both papers it was recognized that both central and C-terminal regions of the two proteins are typical for the WASP family, but their N-termini contained a novel motif. Thus, Miki et al. (1998b) named it WAVE, short for the WASP family verprolin-homologous protein, whereas Bear et al. (1998) dubbed it SCAR because it was identified in a genetic screen for suppressors of the *cAR2*-null phenotype. WAVE/SCAR proteins are sequestered within the WAVE regulatory complex (WRC) that, besides WAVE, also contains the Sra1/Pir121, Nap1, Abi, and HSPC300 subunits [96,97,98,99]. While the activation of WASPs requires direct interaction with Cdc42, WAVE/SCAR proteins do not have a GBD and are regulated by Rac1 indirectly [95]. In an influential publication, it was claimed that the binding of the activated Rac1 to Pir121 in the WAVE1 complex induces the decoupling of the *trans*-inhibitory Pir121-Nap1-Abi2 subcomplex, thus releasing the active WAVE1-HSPC300 heterodimer [97]. Subsequent years have introduced much controversy into this matter, suggesting a constitutive activity of the WRC [100,101,102]. Finally, after realising that experimental conditions could have led to a nonphysiological activation of the WRC, the intrinsic inactivity of different WAVE complexes was strongly confirmed [103,104,105]. However, contrary to the original study of Eden et al. (2002), it was demonstrated that dissociation of the complex does not precede its activation and that Rac1 binding is not sufficient for the activation [105]. Full activation on the membrane requires simultaneous recruitment of the properly phosphorylated WAVE complex by the active Rac1 and acidic phospholipids [105,106].

*D. discoideum* has five members of the WASP family: WASP encoded by *wasA* [107], two unique WASP-related proteins encoded by *wasB* and *wasC* genes [108], SCAR [94], and WASH [109]. *Dictyostelium* WASP has the same domain organization as mammalian WASPs and is an orthologue of the ubiquitous N-WASP [108]. Both *Dictyostelium* WASP and mammalian N-WASP localize to the ventral plasma membrane where they transiently colocalize with clathrin puncta before the onset of the clathrin-coated vesicle (CCV) internalization [110,111,112]. It appears, therefore, that the main physiological role of WASP is to assist in clathrin-mediated endocytosis (CME) in both mammalian and *Dictyostelium* cells [113,114]. WASP is essential for CME in *Dictyostelium* [114], but neither its recruitment to clathrin-coated pits (CCPs) nor its ability to stimulate the Arp2/3-dependent F-actin assembly at the CCPs is dependent on a direct interaction with Rac GTPases (Figure 2A; [115]). CME is the least actin-dependent type of endocytosis in mammalian cells, requiring the support of actin filaments only to overcome high membrane tension or to internalize large cargo [116]. Contrary to the situation in *Dictyostelium*, mammalian N-WASP is not essential for the actin assembly at CCPs since EGFR-mediated endocytosis in N-WASP knockout fibroblasts (devoid of haematopoietic WASP) is not blocked but only reduced [113]. However, an interaction between N-WASP and Cdc42 is required for efficient CME in mammalian cells [117]. Interestingly, both Rho and Rac1 negatively regulate CME [118,119].

In mammalian cells, WASPs are generally required for podosome formation, endocytosis, and phagocytosis, whereas WAVE proteins drive membrane protrusions in the form of lamellipodia and dorsal ruffles [88,120]. Analogously, *Dictyostelium* WASP is not needed for pseudopodia formation [114]. Yet, in addition to clathrin puncta, WASP is also scarcely visible in protrusions of vegetative cells and transiently enriched in the leading edge and the uropod of chemotactically competent cells [107,121]. Therefore, it is not entirely surprising that, in the absence of SCAR, WASP takes over its function by acquiring leading edge localization and driving pseudopodia protrusion to sustain efficient chemotaxis, and this function of WASP requires direct activation by Rac (Figure 2B; [112,115]). On the other hand, *wasA*-null cells exhibit somewhat reduced speed in the chemotaxis to folate due to an inefficient retraction of their enlarged trailing tails that accumulate active Rac and SCAR, which was interpreted as the source of their depolarization [107,114,115]. These authors speculate that WASP functions at CCPs, not only to drive the actin assembly to assist vesicle internalization, but also to remove active Rac from the cell rear via endocytosis, thus maintaining the front-rear polarity [115]. The proposed model therefore depicts WASP as a de facto regulator rather than an effector of Rac. 

Due to the usage of a nonspecific Rac probe that interacts with several active Rac GTPases, including Rac1A/1B/1C and RacC [81], it was not determined which particular Rac GTPases interact with WASP in vivo. WASP GBD interacts with active Rac1, RacA, RacB, and RacC GTPases, but only RacC was shown to be capable of relieving WASP autoinhibition and thus activating F-actin polymerization in vitro and in vivo [82]. Full-length recombinant WASP binds both active Rac1 and RacC [115]. As Rac1 seems to be the major regulator of the leading-edge structures in vegetative cells [122], WASP probably removes Rac1 from the rear, but it is conceivable that other Racs, especially RacC, contribute to front protrusions and/or other WASP-mediated processes. Namely, the phenotype of *wasA*-null cells also implicated WASP activity in phagocytosis, cytokinesis, and starvation-induced aggregation [114], but its regulation in these processes has not been investigated in any detail. Of note, FcγR-mediated phagocytosis in mammalian macrophages requires the Cdc42-induced activation of WASP and N-WASP [123,124].

*Dictyostelium* SCAR (*scrA* gene) is also a part of a heteropentameric inhibitory complex that contains orthologues of PIR121, Nap1, HSPC300, and Abi, each encoded by a single gene [125,126,127]. Although direct interaction between PIR121 and Rac1 (or any other Rac) in *Dictyostelium* has not been demonstrated, microscopy data show that the localization of WRC to active Rac regions at the leading edges of pseudopodia is abolished when wild-type PIR121 is replaced by the Rac1-nonbinding A-site mutant protein [128]. The determination of the structure of the Rac1-bound WRC revealed two Rac1 binding sites in Sra1 (A- and D-site), both required for the WAVE-mediated activation of the Arp2/3 complex [129]. These sites have different binding affinities for Rac1 and distinct functions in vivo, with A-site being a major contributor to the allosteric activation of the complex and crucial for lamellipodia formation [129,130]. The postulated role of Rac in the activation of *Dicytostelium* SCAR is further corroborated by the colocalization of active Rac and SCAR [112,128]. Moreover, the cellular distribution of a probe specific for *Dicytostelium* Rac1 GTPases [131] shows that SCAR localizes to the structures occupied by active Rac1: the leading edges of pseudopodia [112], macropinosomes, and phagosomes [132] and the protruding poles of incipient daughter cells during cell division [133]. Of note, more detailed analysis of macropinocytic and phagocytic cups revealed that Rac is located at the cup centre, while SCAR localizes to the rim of nascent cups [132]. Consistent with their role in F-actin polymerization [134], *scrA*-null cells have a significantly reduced F-actin content [94]. Furthermore, phenotypes of several null mutants generated independently in different genetic backgrounds demonstrate that SCAR plays a role in almost every aspect of cellular motility. Thus, SCAR regulates pseudopod extension and splitting, random cell migration, large-scale endocytosis and endosomal trafficking, cytokinesis, and some aspects of growth and development [94,132,135,136,137].

The best-studied and most prominent evolutionarily conserved role of WAVE/SCAR proteins is the Rac-induced formation of pseudopodia that enables cell migration [95]. Mammalian WAVEs localize to the leading edge of lamellipodia and peripheral ruffles and at the protruding tips of dorsal ruffles [95,138,139,140]. There is ample evidence, obtained from different cell lines, that WAVE proteins play an essential role in the generation of these structures [130,139,140,141,142]. Specifically, the ablation of WAVE, or other components of WRC such as Pir121 and Sra1, induces severe impairment in lamellipodia formation, motility, and directed cell migration, as also observed in Rac-deficient cells [143]. Similarly, *Dictyostelium* cells devoid of SCAR extend significantly fewer pseudopodia, membrane ruffles, and macropinocytic crowns and move at a considerably reduced speed [114,135]. However, the SCAR-deficient cells are capable of generating pseudopodia that support their migration by a WASP-dependent mechanism (Figure 2B; [112,114]). Only the deletion of both SCAR and WASP resulted in immobile cells entirely devoid of pseudopodia [114]. Interestingly, a recent study on acute myeloid leukaemia HL-60 cells showed that haematopoietic WASP also participates in the formation of pseudopodia and is required for the efficient migration of neutrophils [144]. Namely, whereas ubiquitously expressed N-WASP is dispensable for the lamellipodia and filopodia formation in various adherent fibroblast cells [145,146,147], WASP, expressed only in blood cells, is involved in pseudopodia protrusion and migration (Figure 2B; [144,148,149,150,151]). Thus, although being an orthologue of N-WASP, *Dictyostelium* WASP also shares functions with a mammalian N-WASP paralogue, the haematopoietic WASP, which emerged at the onset of vertebrate evolution [108].

Interestingly, *Dictyostelium* and mammalian cells share another Rac1 interactor, CYRI (CYFIP-related Rac interactor), which is a negative regulator of SCAR/WAVE activity [152]. CYRI is an evolutionary conserved protein that binds active Rac1 via its DUF1394 domain and competes with SCAR/WAVE for active Rac1 at the leading edge. Thus, CYRI locally buffers Rac1 activity and thereby inhibits the SCAR/WAVE-induced cell protrusions to achieve optimal actin dynamics for efficient cell polarization and steering during cell migration [152]. CYRI is also required to establish Rac1-dependent polarity during epithelial cyst formation [152,153].

#### 4.1.2. Formins as Rac1 Effectors

An early study has indicated that *Dictyostelium* Rac1 GTPases play a pivotal role in the protrusion of filopodia [76]. It was soon shown that this function is mediated through another actin nucleator, the Diaphanous-related formin H [23]. Indeed, the WASP family of NPFs are dispensable for the protrusion of filopodia in *Dictyostelium* and mammalian cells alike [114,135,139,145,147,154]. Formins constitute a distinct class of actin nucleators that, unlike the Arp2/3 complex, promote de novo nucleation of unbranched filaments and their rapid elongation, acting as processive actin polymerases [120]. Formins are characterized by the presence of an FH2 domain that is necessary and sufficient for actin assembly [155]. In most formins, the FH2 domain is preceded by an FH1 domain that accelerates actin polymerization by channelling the ATP-G-actin subunits [156,157]. For a detailed overview of formin-mediated actin assembly mechanisms the reader is referred to a recent review [158]. Diaphanous-related formins (DRFs) are a subgroup of formins characterized by additional regulatory elements flanking the FH1 and FH2 domains, N-terminal GBD and FH3 domains where FH3 contains a Diaphanous-inhibitory domain (DID), a dimerization domain (DD), and a C-terminal Diaphanous-autoregulatory domain (DAD) [159,160]. These regulatory domains enable a direct regulation of DRFs by Rho GTPases [161]. In its inactive state, DRF is autoinhibited by an intramolecular interaction between DAD and DID, and the binding of a Rho GTPase to GBD, in the presence of an additional cofactor, relieves this inhibition.

The *D. discoideum* formin family is comprised of ten proteins, ForA to J, and eight thereof belong to DRFs [162]. ForH is a canonical DRF with the typical GBD/FH3-FH1-FH2-DAD domain organization and exerts typical formin activities [23]. ForH strongly binds to activated Rac1A in vivo, localizes to the tips of growing and mature filopodia, and is specifically required for the extension of filopodial actin filaments [23]. ForH is an orthologue of a mammalian DRF, mDia2, hence its alternative name, dDia2 [23]. The roles of mammalian DRFs, notably mDia1 and mDia2, in generating long linear actin filaments in filopodia are well established [163,164,165,166,167]. mDia2 is required for the formation of filopodia downstream of two Rho GTPases, Cdc42, and Rif (RhoF). In addition to binding to RhoA–C, which are the major regulators of mammalian formins [161,168], mDia2 also binds the active Cdc42 via a CRIB motif in its GBD domain [169,170]. It localizes to the tips of Cdc42-induced filopodia and is required for Cdc42-induced filopodia formation [170]. In addition, mDia2 participates in a Cdc42-independent mechanism of filopodia formation that involves the small GTPase Rif (Rho in filopodia) and localizes to the tips of Rif-induced filopodia (Figure 2C; [171,172]).

#### 4.1.3. Coronins as Rac1 Interactors

Coronins are another class of Rac interactors found in both *Dicytostelium* amoebae and mammalian cells. Coronins inhibit the Arp2/3-mediated actin filament nucleation directly by binding to Arp2/3 complex subunits [173]. They also indirectly promote the filament turnover in lamellipodia by regulating cofilin activity via Slingshot phosphatase and by antagonizing the activity of cortactin, a filament branch stabilizer [173,174,175]. Mammalian coronins are divided into three types, where types I (coronin 1A, 1B, 1C and 6) and II (coronin 2A and 2B) share the same basic structural organization containing one WD-repeat domain that folds into a β-propeller structure, while type III coronin 7 contains two β-propeller units in tandem [175]. The *Dictyostelium* genome codes for two coronins: conventional type I-like coronin (encoded by *corA*), the founding member of the coronin protein family, and coronin 7 (encoded by *corB*), an orthologue of human coronin 7 [176,177]. In *D. discoideum* cells, coronin is involved in the regulation of phagocytosis, cytokinesis, cell polarization, motility, chemotaxis, and initiation of development [178,179,180,181]. It localizes to the phagocytic cups and the crown-shaped dorsal projections of growth-phase cells, i.e., the macropinosomes, and to pseudopodia of both vegetative and aggregation competent cells [176,179]. It is also enriched at the polar regions of nascent daughter cells during cell division but is excluded from the cleavage furrow [178]. Coronin 7 is also involved in phagocytosis, chemotaxis, and development and localizes to the leading front of both vegetative and aggregating cells [177,180]. It is also recruited to nascent phagocytic cups, with enrichment at its tips, and persists there until completion of the particle ingestion [177].

Coronin family members contain a CRIB motif, though it is only moderately conserved in *Dictyostelium* coronin 7 [121,182]. Nonetheless, direct interactions with Rac GTPases were demonstrated for both *Dictyostelium* coronins, but they preferentially bound to the GDP-loaded Rac GTPases; coronin to the Rac1 GTPases, RacB and, somewhat more weakly, RacC; and coronin 7 to RacA, RacC, RacE, and, weakly, to Rac1 GTPases [85,121]. These interactions appear to have a role in sequestering Rac and thus regulate the balance between active and inactive Rac GTPases in the cell. Based on increased levels of activated Rac GTPases and myosin II filaments in *corA*-null cells, a model was proposed wherein coronin regulates the Rac activity and prevents it from overly activating its downstream effector PAKa that promotes myosin II assembly by inhibiting myosin II heavy chain kinases (MHCKs) [85]. Apparently, coronin can also directly bind PAKa and regulate its activity [85]. A higher binding affinity towards GDP-bound Rac1 and a Rac-sequestering role was also demonstrated for mammalian coronin 1C [183]. Coronin 1C interacts with GDP-bound Rac1 and RCC2, a negative regulator of Rac1 activity, and prevents the mislocalized activation of Rac1. Coronin 1C redistributes inactive Rac1 from the lateral to the front membrane, where RCC2, which also binds Rac1 directly and prevents its activation by GEFs, takes on a sequestering role as long as activation signals are absent. Upon sufficient local activation of GEF, RCC2 becomes outcompeted, and local Rac1 activation leads to the formation of a single dominant protrusion [183]. The depletion of coronin 1A or 1B induces the formation of myosin II-dependent structures similar to Dictyostelium *corA*-null cells; however, both mammalian coronins are important for Rac1-induced cytoskeletal changes via the Rac1-ArhGEF7-Pak2 signalling pathway [184]. Interestingly, and in contrast to *Dictyostelium* coronin, coronin 1A favours Rac1 activation by promoting its translocation to the plasma membrane [185]. Coronin 1A forms a complex with PAK1 and RhoGDIα that facilitates the release of Rac1 from the RhoGDIα inhibitory grip via the Pak1-dependent phosphorylation of RhoGDI. Formation of the coronin 1A-Pak1-RhoGDIα complex is dependent on the coronin 1A interaction with the catalytically inactive ArhGEF7 and F-actin [185].

In contrast to *corA*-null cells, there was no significant increase in Rac activity in *corB*-null cells, which could be explained with a higher amount of coronin in the cell and its substantially more efficient binding to Racs compared to coronin 7 [121]. Still, the authors proposed that coronin 7 restricts the local activation of Rac in the regulation of phagocytosis and motility. Namely, WASP was found in a complex with coronin 7 and the expression of GFP-WASP in *corB*-null cells reverted a significantly increased uptake of yeast particles to wild-type levels. It was inferred, therefore, that coronin 7 regulates actin depolymerisation and WASP activation at the phagocytic cups. The same was suggested for SCAR in the coronin 7-mediated regulation of motility and development [121].

Ubiquitously expressed mammalian coronin 7 localizes to the Golgi apparatus and is crucial for the maintenance of the Golgi architecture and anterograde trafficking [186,187,188]. Similar to its *Dictyostelium* counterpart, mammalian coronin 7 preferentially binds to the GDP-loaded Cdc42 and considerably less to the GDP-loaded Rac1, and this interaction is necessary to support the Golgi structure, presumably by regulating the activity of the Golgi-associated Cdc42 [186]. Unexpectedly, the level of activated Cdc42 was markedly reduced in knockout cells, suggesting that the Golgi-associated coronin 7 activates the subpopulation of Cdc42 that regulates the Golgi apparatus structure. On the other hand, coronin 7 was shown to directly interact with the Cdc42 target N-WASP, thus suppressing the N-WASP-promoted formation of excessive actin filaments which could harm the integrity of the Golgi apparatus [186].

#### 4.1.4. PAK Kinases as Rac1 Effectors

p21-activated kinases (PAKs) are serine/threonine protein kinases that act as effectors of small GTPases and regulate the actin cytoskeleton via phosphorylation and interaction with numerous proteins involved in cytoskeletal rearrangements, motility, cell cycle, and survival [189,190]. PAKs can be found from protozoa to mammals, and all have a N-terminal regulatory GBD (a.k.a. PBD—p21 binding domain) that contains the CRIB motif and a conserved C-terminal kinase catalytic domain [190,191]. The six PAK isoforms in humans are divided into two groups [189]. Group I PAKs (1–3) harbour an autoinhibitory domain (AID) at the N-terminal region that, following a GTPase-dependent or independent activation, separates from the kinase domain, allowing the autophosphorylation of PAK [189,190,191]. Interaction with Rac/Cdc42 leads to the translocation of group II PAKs to different cellular compartments where they phosphorylate the target proteins [190,191,192].

The Rac/Cdc42-mediated activation of PAK1 induces the formation of lamellipodia and filopodia during directional cell migration by regulating F-actin localization at the leading edge of the cell [189,191,193,194]. Furthermore, Rac/Cdc42-activated PAK1 and PAK2 regulate the actin–myosin contractility by phosphorylation and inhibition of the myosin light chain kinase (MLCK) activity [195,196]. PAK1 and PAK2 also participate in a positive feedback loop by phosphorylating RhoGDI1, which leads to the activation of Rac1 and Cdc42 [197,198]. PAK3 and 4 are known to preferentially bind Cdc42 rather than Rac [192]. PAK3 activated by Cdc42 phosphorylates paxillin α is involved in the formation of integrin-dependent focal adhesions [199]. PAK4 activation by Cdc42 regulates the formation of filopodia [200].

*D. discoideum* genome contains nine genes encoding eight PAKs that can be divided into two groups based on the similarity of their catalytic domains: PAKa–d and PAKe–h [35]. PAKa, PAKb, PAKc, and PAKd play roles in chemotaxis, cell polarity, and endocytosis, but an interaction with Rho GTPases was shown only for the first three [81,84]. PAKa is expressed during vegetative and early developmental stages, with a maximum during aggregation. PAKa localizes to the rear of migrating cells and *pakA*-null cells show diminished directionality during chemotaxis, suggesting that PAKa plays a role in the suppression of lateral pseudopodia and retraction of the cell tail [201,202]. An initially indicated involvement of PAKa in cytokinesis [201], however, was later disputed [202,203]. Interaction studies showed that PAKa interacts in vitro with active forms of Rac1A/B/C, RacB, RacA, and human Cdc42 and Rac1, as well as with PKBa, coronin, and 14-3-3 protein [84,85,202,204]. Interestingly, coimmunoprecipitation and pull-down assays showed that coronin interacts with PAKa through its CRIB motif (see Section 4.1.3; [85]). cAMP-mediated PAKa activation and translocation to the cell rear is regulated via a direct phosphorylation by PKB/Akt, as opposed to an indirect activation of mammalian PAK1 through Akt [202,205]. PAKa regulates the myosin II assembly by inhibiting MHCKs A and C or by activating MHC phosphatase [85,201,206]. 

Although no clear functional significance of the PAKa interaction with Rac GTPases has been deciphered yet, its GBD was successfully used to track and quantify the spatio-temporal activity of Rac1 in *Dictyostelium* cells [131]. Y2H, pull-down, and fluorescence resonance energy transfer (FRET) assays showed that PAKa GBD specifically interacts with the active Rac1A, and the fluorescently labelled probe localizes to the leading edge of migrating cells and to endocytic cups [131]. In comparison to previously used fluorescent Rac-GTP probes based on the GBDs of PAKb [81,112] and mammalian PAK1 [122], the PAKa GBD probe produces much less background, and its expression level also remains adequate during aggregation and introduces no overexpression artefacts [131].

PAKb was first purified as a myosin I heavy chain kinase (MIHCK) responsible for myosin ID (MyoD) activation [207]. Furthermore, Y2H, pull-down, and coimmunoprecipitation assays showed that, besides MyoD, PAKb binds MyoK and the actin-binding protein 1 (Abp1) [207,208,209]. PAKb increases the motor activity of MyoK and MyoD by phosphorylating their TED sites [207,208]. It was suggested that MyoK and PAKb together with Abp1 form a loop to regulate phagocytosis [208]. PAKb localizes to the leading edge of migrating cells and to phagocytic and macropinocytic cups by its proline-rich N-terminal region, which largely coincides with the localization of several myosin I proteins [81]. Interestingly, however, no specific significant defects in any of the myosin I-dependent processes could be detected in cells lacking PAKb, but they have a mild chemotaxis defect, suffer from the loss of polarity, and produce superfluous lateral pseudopodia [81,83]. PAKb binds to actin filaments by its actin filament-binding module at the N-terminal part of the protein, and it was suggested that a PAKb-Abp1 complex has a role in the cross linking of actin filaments [209]. A Y2H assay showed that the PAKb PBD binds the active Rac1A/B/C, F1, B, C, and GTPase domain of RacA [81] Interestingly, human Cdc42 and Rac1, but not RhoA, also interact with PAKb and stimulate its activity, indicating a conserved regulatory mechanism [210]. Acidic phospholipids can stimulate the PAKb autophosphorylation and kinase activities as effectively as active Rac1, but this activation mechanism becomes inhibited by the binding of Ca^2+^-calmodulin to PAKb [211]. 

Similar to PAKa and PAKb, PAKc is required for proper chemotaxis since *pakC*-null cells exhibit a loss of polarity and produce excessive lateral pseudopodia [83]. Cells lacking both PAKb and PAKc have an even stronger chemotaxis defect with a greatly reduced speed, suggesting that PAKb and PAKc may have multiple overlapping functions. The overexpression of PAKc in wild-type cells also leads to a reduced speed and directionality [83]. A rapid, transient activation and translocation of PAKc from the cytosol to the plasma membrane occurs upon chemoattractant stimulation. In addition to the typical PAK domains, PAKc also contains an N-terminal pleckstrin homology (PH) domain interacting primarily with PI(3,4)P2 and a C- terminal extension related to the Gβγ binding domain of the *Saccharomyces cerevisiae* Ste20 kinase and mammalian PAK1 [83]. It was shown that the PAKc GBD interacts with the constitutively active RacB, point mutations in the CRIB motif cause the loss of binding to active RacB and abolish PAKc activation, and *racB*-null cells exhibit no activation of PAKc, jointly suggesting that RacB is required for chemoattractant-mediated PAKc activation (see Section 4.2; [83,84]). It was also shown that the proper PAKc activation and translocation to the plasma membrane in vivo requires all its domains [83]. 

PAKd is involved in the regulation of actin dynamics in both vegetative and aggregating cells [212,213]. During cell migration, PAKd localizes to a single punctum within the cell and occasionally to the rear cortex in vegetative cells [212], whereas it translocates to pseudopods and uropods in response to cAMP gradients [213]. Consistently, *pakD*-null cells exhibit defects in chemotaxis to cAMP and are unable to form proper aggregates upon starvation [213]. PAKd is also important for the inhibition of cell proliferation at high cell densities and for growth-to-differentiation signalling [212,214,215]. PAKd has not yet been investigated in the context of regulation by Racs.

#### 4.1.5. IQGAP-Related Proteins as Rac1 Effectors

IQGAP (*IQ*—IQ domain; *GAP*—domain with sequence similarity to the catalytic domain of RasGAPs, also known as GRD, GAP-related domain) proteins are well-established effectors of both mammalian and *Dictyostelium* Rho GTPases. Initially, they were considered to function as RasGAP proteins, but early experiments demonstrated that they do not bind Ras GTPases or have a RasGAP activity [216,217,218,219,220]. Instead, mammalian IQGAP1 and IQGAP3 interacted with activated Cdc42 and Rac1, with a higher affinity for Cdc42 [217,218,219,221], while IQGAP2 interacted with these two GTPases without discriminating their GTP- and GDP-bound forms [216,219]. Furthermore, it was demonstrated that not only do IQGAPs lack a GAP activity towards any small GTPase, but they also can stabilize Cdc42 and Rac1 in their active states by inhibiting their intrinsic and RhoGAP-stimulated GTPase activity [216,217,219].

IQGAP proteins are evolutionarily conserved proteins that have three family members in the majority of vertebrates [222]. These are relatively large proteins (human IQGAPs have around 1600 amino acid residues) containing several domains and motifs that enable them to interact with various binding partners and act as scaffolds to integrate and modulate different signalling pathways [223]. All three isoforms share the same domain organization from the N- to the C-terminus: a single F-actin-binding CHD [224,225,226], an IQGAP-specific repeat responsible for dimerization [226], a WW motif that binds ERK1/2 [227,228], a calmodulin-binding IQ domain [217,229,230], a GRD that binds Cdc42 and Rac1 [217,231,232], and a RGCT domain unique to IQGAPs that binds β-catenin, E-cadherin, and CLIP-170 [233,234]. Here, we have included only a subset of prominent IQGAP-interacting proteins; for an exhaustive list the reader is referred to recent reviews [222,235]. Mammalian IQGAPs participate in diverse cellular processes; they cross link actin filaments, regulate actin dynamics and actin-microtubule crosstalk, and are involved in the regulation of higher order processes, such as cell polarization and directional migration, cytokinesis, cadherin-mediated cell–cell adhesion, vesicle trafficking, intracellular signalling, cell proliferation, and gene expression [223,235,236,237,238,239].

*D. discoideum* has four IQGAP-related proteins: DGAP1/DdIQGAP1, GAPA/DdIQGAP2, IqgC/DdIQGAP3, and IqgD/DdIQGAP4. They are half the size of mammalian IQGAPs (817–860 amino acids), aside from IqgD, which is larger (1385 amino acids). Basically, they are homologous to the C-terminal half of mammalian IQGAPs containing the juxtaposed GRD and RGCT domains [240]. Since they lack the N-terminal half of mammalian IQGAPs, *Dictyostelium* IQGAPs cannot bind F-actin directly. The only exception is IqgD, which harbours a CHD duplex at its longer N-terminus [241], but IqgD still awaits functional characterization. Of the three studied IQGAP-related proteins, DGAP1 and GAPA show features of typical IQGAP family members [242,243], while IqgC is not a typical IQGAP. IqgC has conserved residues in its GRD domain critical for the GAP activity that are mutated in other IQGAPs, and it was demonstrated to be a genuine RasGAP that binds to and inactivates RasG [244,245]. DGAP1 and GAPA interact with Rac GTPases and do not have a GAP activity, but, as opposed to their mammalian counterparts, they cannot stabilize the GTP-forms of bound GTPases [76,243,246,247]. 

All three *Dictyostelium* Rac1 GTPases interact strongly with DGAP1 [76], and it was shown that DGAP1 recruited by activated Rac1A promotes the formation of a tetrameric complex with the cortexillin I (CI)/cortexillin II (CII) heterodimer [246]. Cortexillins are actin-binding proteins that organize actin filaments preferentially into antiparallel bundles, associate them into meshwork, and stabilize cell shape by supporting the intrinsic stiffness of the cell cortex [248,249]. Subsequently it was demonstrated that all three Rac1 GTPases can participate in a complex formation with either DGAP1 or GAPA, and yet another cortexillin III was identified in a complex with DGAP1 but not GAPA [250]. Therefore, both DGAP1 and GAPA activated by Rac1 can promote complex formation, but in the absence of both proteins, the complex cannot be assembled [246]. DGAP1, GAPA, CI, and CII localize to the lateral and rear parts of the cortex in interphase cells and to the cleavage furrow in dividing cells [122,246,247,248]. Moreover, activated Rac1 can be seen in the cortex of the incipient cleavage furrow during cytokinesis, although it is significantly enriched at the polar regions of the nascent daughter cells [131]. The localization of active Rac1 during cytokinesis is highly reminiscent of the localization of another Rac1-binding partner, filamin, which, interestingly, binds both Rac1 and GAPA (see Section 4.1.6; [247]).

Phenotypes of single and double knockout mutants of the two IQGAPs and the two cortexillins have demonstrated the crucial role of their quaternary complexes for efficient cytokinesis [242,246,248]. They also play a role in the establishment of cell polarity and motility during chemotaxis by supressing lateral pseudopodia [250], and in the intercellular cAMP signal relay, which is important for cell streaming and development [251]. In late development, the DGAP1-CI-CII complex is involved in securing the apical localization of myosin II in the tip epithelial cells surrounding the stalk tube, thus promoting apical actomyosin ring constriction to ensure normal culmination of the fruiting body [252].

Drawing a parallel between the actin-targeted functions of mammalian IQGAPs and *Dictyostelium* IQGAP-related proteins is not straightforward. Besides binding directly to and crosslinking actin filaments, mammalian IQGAP1 also promotes actin filament assembly via its interactions with N-WASP, the Arp2/3 complex, and formin mDia1 [253,254,255]. *Dictyostelium* DGAP1 and GAPA do not directly bind F-actin, and no interactions with NPFs or actin polymerases have been reported. In line with these data, IQGAP1 localizes to the leading edge of polarized cells and to membrane ruffles [217,218], whereas DGAP1 and GAPA are mostly confined to the rear and lateral structures in polarized cells [122,247]. However, Rac1-bound DGAP1 and GAPA interact with F-actin-binding cortexillins and thus indirectly promote F-actin bundling similar to mammalian IQGAPs. It is, therefore, still possible to compare the final functional outputs of the signalling pathways mediated by mammalian IQGAPs and *Dictyostelium* IQGAP-cortexillin complexes.

Both *Dictyostelium* and mammalian IQGAPs are required for cytokinesis completion [236,242,246]. The knockdown of IQGAP1 or IQGAP3 moderately increased the number of multinucleated HeLa cells, while the simultaneous knockdown of both proteins induced a more severe multinucleated phenotype, similar to the cumulative effect of the *dgap1/gapA* double knockout [236,246]. IQGAP3 accumulates in the contractile ring region where it interacts with the scaffold protein anillin, while IQGAP1 retains its cortical localization throughout cytokinesis. IQGAP1 and IQGAP3 thus appear to have nonredundant roles in cytokinesis, similar to the nonredundant roles ascribed to DGAP1 and GAPA [246]. Interestingly, the downregulation of RhoA, but not Cdc42, inhibited the proper localization of IQGAP3. Reciprocally, RhoA accumulation in the cleavage furrow was significantly reduced and/or disturbed in the absence of IQGAP1 and IQGAP3. However, neither of the IQGAPs was shown to interact with RhoA directly [217,218,221,231,236].

Maintenance of cell polarity during the directed cell migration involves an asymmetric distribution of signalling molecules and cytoskeletal components. *dgap1*-null cells show a mild, and *gapA*-null cells a moderate, decrease in the ability to supress lateral pseudopodia, whereas the *dgap1/gapA* double knockout cells exhibit markedly decreased migration speed, directionality, and cell polarity [250]. This effect was attributed to disturbed cortical mechanics and highly elevated and extended PI3K/PKB signalling in the cortex of mutant cells. Thus, the *Dictyostelium* DGAP1-cortexillin complex is involved in the establishment of cell polarity by restricting the leading-edge functions to the cell front. Similarly, mammalian IQGAP1 is involved in the regulation of cell polarity via the microtubule plus-end binding protein CLIP-170. Activated Rac1 or Cdc42 form a tripartite complex with IQGAP1 and CLIP-170, and IQGAP1 serves as a linker between the actin cytoskeleton and the microtubules, helping to capture microtubules at the leading edge and the base of filopodia, thus facilitating the establishment of a polarized cell morphology [233].

Besides its involvement in the regulation of cell polarity during migration, the DGAP1-cortexillin complex is also required for the apico-basolateral polarity of the tip tubular epithelia during multicellular development [252]. α-catenin together with DGAP1 and cortexillins act to exclude myosin II from the basolateral membranes and ensure its apical localization, where an actomyosin ring responsible for apical constriction is assembled. Such epithelial tubes, with apical membranes facing the lumen, are found in many tissues in our body and use apical actomyosin constriction to oppose luminal pressure [256]. Although direct evidence for an IQGAP-mediated myosin II exclusion from the basolateral membranes in animal cells is missing, IQGAP1 is still important for basolateral polarity during epithelial tube morphogenesis. In mitotic cells, IQGAP1 localizes to basolateral membranes, and this EGFR-mediated polarized distribution of IQGAP1 is required for anchoring of the astral microtubules to junctional plasma membranes and a correct orientation of the mitotic spindle during epithelial tube formation [257].

#### 4.1.6. Filamins as Rac1 Effectors

As already mentioned, Rac1 interacts with the F-actin binding protein filamin [247]. Filamins are cross linking proteins that orthogonally connect actin filaments to stabilize their three-dimensional network, which provides elastic and strain-stiffening properties to the cell [258,259]. Two actin-binding domains (ABDs) required to connect neighbouring filaments are provided by the dimerization of two self-associating filamin subunits. Filamins also act as scaffolds that, e.g., link the actin cytoskeleton to membrane receptors and facilitate the colocalization of various signalling proteins [258,260].

Being substantially shorter than mammalian filamins, *Dictyostelium* filamin, ddFLN (or actin binding protein C (ABP120), or gelation factor) is considered to represent an ancestral form of human filamins [241,259]. It is involved in the pseudopod formation and motility in response to cAMP stimulation and in phagocytosis and cytokinesis [247,261,262,263]. It is also essential for the normal phototaxis and thermotaxis of slugs during multicellular development [264]. *Dictyostelium* filamin interacts and, for the most part, colocalizes with GAPA at the cortex of interphase cells and partially rescues the phenotype of *gapA*-null cells [247]. Although it is found in the complex with GAPA and Rac1A, filamin can also interact with Rac1A in the absence of GAPA, and it was suggested that it acts as a scaffold for Rac1-GAPA signalling to the actin cytoskeleton [247].

Similar scaffolding roles in Rac1 signalling have been described for mammalian filamins A and B [265,266,267,268]. For example, filamin A is involved in the integrin-mediated Rac1 deactivation to constrain its activity during cell migration [266]. Filamin A and IQGAP1 are recruited to the activated β1 integrin in early adhesion structures in lamellipodia, and subsequently, the filamin A-IQGAP1 complex recruits RacGAP1 to suppress Rac1 activity [266]. Filamin B is implicated in the regulation of endothelial cell migration and angiogenesis [265]. In a basal state, Rac1 and its GEF Vav2 are in a complex with filamin B, and following VEGF stimulation, VEGFR2 and proangiogenic integrin αvβ5 are recruited to the complex. This complex built around filamin B appears to modulate both Rac1 activity and its intracellular localization [265].

### 4.2. RacF1, RacF2, and RacB

RacF1 and RacF2 proteins are expressed throughout the asexual *Dictyostelium* life cycle and also during sexual development induced under dark and submerged conditions [75,269]. In particular, the gene for RacF2 shows extremely high expression levels in gametes during sexual reproduction. RacF2 affects sexual cell fusion and, presumably, also asexual development by regulating EDTA-sensitive cell–cell adhesion. RacF1 is enriched at the plasma membrane, pseudopodia, early macropinosomes and phagosomes, and the sites of cell–cell contacts [269]. However, *racF1*-null, *racF2*-null, and double knockout cells do not show any serious defects in the vegetative phase or asexual development, suggesting that they are dispensable, or their activity is compensated for by other Racs [75,78,269]. Since RacF1 and RacF2 are 94% identical, they are likely to have redundant roles in sexual cell fusion.

Similar to RacF1 and RacF2, RacB from *D. discoideum* is closely related to the Rac1 group (Figure 1) but is mostly expressed during the vegetative phase and in early development [72]. RacB has been shown to play a role in endocytosis, chemotaxis, and morphogenesis [84,270]. Cells with overexpressed wild-type RacB have a flattened and round morphology, significantly reduced fluid phase uptake, slightly reduced phagocytosis, and exocytosis and, over time, show signs of lysis, suggesting that high levels of RacB could be toxic [270,271]. The kinetics of the cAMP-stimulated RacB activation closely correspond to the kinetics of actin polymerization, and *racB*-null cells exhibit significantly reduced chemoattractant-induced polymerization peaks of F-actin, suggesting that RacB is involved in this process [84]. *racB*-null cells also show significantly diminished chemoattractant-induced myosin II assembly; show no activation of PAKc; show a chemotaxis defect with reduced speed, polarity, and directionality; and have a significant developmental delay [84].

Interaction studies using Y2H and pull-down assays identified GAPA, PAKa, PAKb, PAKc, WASP, and WASP-B as binding partners of RacB-GTP [80,81,82,83,84,247]. It was shown by these assays that activated RacB strongly interacts with the CRIB motifs of PAKa and PAKc, which might explain the diminished myosin II assembly in *racB*-null cells, since PAKa is known to be required for the assembly of myosin II filaments [84]. Additionally, point mutations in the PAKc CRIB abrogate its binding to RacB-GTP, which leads to chemotaxis defects similar to the reduced polarization and directionality of *pakC*-null cells [83]. It has been suggested that RacB and RacC are involved in the regulation of actin polymerization by competing for interaction with the WASP CRIB [82]. It has also been suggested that coronin is involved in the sequestration of RacB, similar to the coronin-mediated Rac1 sequestration (see Section 4.1.3; [85]).

### 4.3. RacA

RacA is expressed throughout the vegetative phase and development and reaches the maximum level of expression after 12 h of starvation [72,272]. In addition to the G1–G5 and the two switch regions, RacA has a proline-rich region, two BTB (broad-complex, tamtrack, bric à brac) domains, and a specific C-terminal region, which are all characteristic for the mammalian RhoBTB subfamily GTPases (Figure S1). Similar to mammalian RhoBTB1 and RhoBTB2, RacA also lacks the CAAX prenylation motif and is twice as large as other Rho GTPases from *D. discoideum*. It has therefore been suggested early on that RacA should be considered as a member of the RhoBTB subfamily [272]. Among the human RhoBTB GTPases, RhoBTB1 and RhoBTB2 are closely related to each other, whereas RhoBTB3 lacks most parts of the GTPase domain and has the conserved CAAX motif, but the structure of its BTB1 domain is more similar to RacA [273]. All three human RhoBTB proteins are considered to be tumour suppressors [274]. BTB domains are involved in protein–protein interactions and have a role in the formation of cullin3-dependent ubiquitin ligase complexes, thus possibly targeting proteins for degradation [275,276]. According to the data reported as preliminary, *racA*-null cells have a severe growth defect, which is apparently not a consequence of impaired cytokinesis or endocytosis [59]. Interaction studies using a Y2H assay identified PAKa, PAKb, PAKc, WASP, and WASP-B as binding partners of RacA-GTP, which are also effectors of Rac1 and RacB, and some of them also for RacC and RacF1 [80,81,82,84]. An interaction study by a pull-down assay also identified GAPA as a possible binding partner of RacA [247].

### 4.4. RacC

Our phylogenetic analysis shows that the amoebozoan RacC group, identified in all analysed amoebozoan classes, represents a sister clade to the metazoan group consisting of Cdc42 and a number of related GTPases including human RhoJ, Q, U, and V (Figure 1 and Figure S3; [277,278,279]). Functional data regarding the RacC from *D. discoideum* show that its roles in the regulation of the actin cytoskeleton are interwoven with the roles of Rac1 and other Rac subfamily GTPases in an intricate way, somewhat resembling a partial overlap between the roles of Cdc42 and Rac GTPases in mammals (Figure 2; [79]). Cdc42 is traditionally best known for its involvement in filopodia formation [280], acting predominantly through DRFs [161,170], but also by activating the Arp2/3 complex through N-WASP [91]. Over time, it was realized that Cdc42 is involved in many different aspects of cell polarity by regulating the formation of cytoskeletal structures [281,282] and thus also influences the cellular migration and invasion processes underlying tumour formation [283]. Active Cdc42 thus regulates cell polarity in migrating cells via MRCKs (myotonic dystrophy-related Cdc42-binding kinases), which regulate the actin–myosin contraction involved in the reorientation of the cell nuclei relative to the microtubule-organizing centres [284], and by activating the PKCζ-mPar6 complex [285]. The recruitment of mPar6 and PKCζ (protein kinase C zeta) by Cdc42 leads to the GSK-3β phosphorylation at the leading edge, inducing association of the Apc protein with microtubules, which is essential for cell polarization [286]. 

*D. discoideum* RacC shows a steady expression during growth and development [72,73,74]. RacC was shown to associate with the plasma membrane in vegetative cells [287,288], and to accumulate in the areas of F-actin assembly at the leading edge of chemotactically competent cells [80,82]. It was also found on cytoplasmic vesicles in both vegetative [122] and aggregation competent cells [82]. Similarly, Cdc42 was shown to partially localize to the plasma membrane, but a significant fraction was associated with Golgi vesicles [289]. *racC*-null cells exhibit motility defects during chemotaxis due to a lack of polarized F-actin organisation and a well-defined leading edge [82]. In addition, they also have a cytokinesis defect [78,82] but show no significant alterations of the Golgi morphology in comparison to wild-type cells [69]. The overexpression of wild-type RacC induces the formation of irregular actin structures on the cell dorsal surface, named petalopodia [288]. It also strongly upregulates phagocytosis, while inhibiting macropinocytosis and exocytosis [288]. While the deletion of RacC leads to defects in both speed and directionality during chemotaxis of *Dictyostelium* cells, the effects of Cdc42 deletion are cell-type specific. For example, *cdc42*-null mouse embryonic fibroblasts (MEFs) are impaired both in motility and directionality during chemotaxis [290], while some cancer cells still migrate with normal speed, only randomly [291].

Cdc42 is able to activate more than 45 effector/adaptor proteins encoded in the human genome, including PAKs, IQGAPs, N-WASP, PI3Ks, and others [282,283,292]. Interaction studies identified WASP [82,115], WASP-B [80], coronin [85], PAKc [84], and PAKb [81] as binding partners of RacC, but only interactions with WASP and WASP-B have been further investigated. As already mentioned, RacC activates WASP and promotes F-actin assembly in a PI3K-dependent manner (see Section 4.1.1; [82]). On the other hand, RacC is required for PI3K activation and translocation to the plasma membrane since both basal and chemoattractant-stimulated levels of PI3K at the plasma membrane are decreased in *racC*-null cells [82]. Besides WASP, *Dictyostelium* expresses two additional proteins from the WASP group, WASP-B and WASP-C [108]. Although their domain topology is unconventional, they both contain WCA and GBD domains and, like WASP, localize to clathrin puncta, suggesting an involvement in CME [108]. Furthermore, *wasB*-null cells are defective in chemotaxis due to a lack of polarized F-actin distribution, leading to the formation of pseudopodia at the rear and lateral parts of the cells [80]. This phenotype is highly reminiscent of a diminished cell polarity observed in *wasA*-null cells [114]. Interestingly, RacC activation is increased but RacC is mislocalized in the absence of WASP-B. Of note, WASP-B GBD also interacted with constitutively active Rac1B, RacA, and RacB, but the functional significance of these interactions was not further examined [80]. WASP-C is implicated in the regulation of actin cytoskeleton, the cell-substratum adhesion, and phagocytosis, but its regulation has not been investigated so far [293].

Adenylyl cyclase A (ACA) is a RacC interactor that is responsible for cAMP synthesis and localizes to the uropod during stream formation [69,294,295]. Since *racC*-null cells are defective in stream formation and ACA in these cells is found at the intracellular vesicles instead of on the plasma membrane, it was inferred that RacC is important for ACA vesicle trafficking [69]. Similar to this potential role of RacC in vesicle trafficking, Cdc42 is involved in vesicle formation and trafficking, which was shown to be mediated by the regulation of actin dynamics through N-WASP and Arp2/3 [296,297]. RacC was also shown to be essential for the autocrine proliferation repressor AprA-induced chemorepulsion [298,299]. Vegetative cells continuously secrete AprA, and its secretion from the cells at the colony edge signals to adjacent cells to move away [299]. Specifically, AprA inhibits the formation of new pseudopodia at the side of the cells closest to the colony. The *racC*-null cells were unable to respond to this chemorepellent [299].

### 4.5. RacH, RacD and RacP

In the phylogenetic tree, RacH, D, and P from Dictyostelia occupy a position between the Cdc42/RacC group and the Rho/RacE group but are more closely related to the latter (Figure 1). Lacking any obvious homologues in Metazoa, these proteins are also abundantly represented in Archamoebae, and can thus be regarded as a truly amoebozoan innovation. 

*Dictyostelium* RacH is expressed throughout the life cycle, with the highest level in the aggregation stage [272,300]. It localizes to the nuclear envelope, endoplasmatic reticulum, and Golgi membranes [301]. *racH*-null cells grow moderately slower in suspension and show impaired macropinocytosis and a slight decrease in the acidification of early endosomes [301]. Their exocytosis efficiency is also reduced, probably due to an aberrant distribution of vacuolin [301]. Therefore, it has been proposed that RacH acts as a regulator of vesicle sorting. Furthermore, RacH also plays an important role in host–pathogen interactions, as it was shown that *racH*-null cells are more susceptible to infection by *Mycobacterium marinum* or *Legionella pneumophila*: the bacteria proliferate more easily in *racH*-null cells, which was attributed to a defect in endosome acidification [302,303]. On the other hand, the depletion of RacH has a deteriorating impact on bacterial transmission between *Dictyostelium* cells, since the release of bacteria from *racH*-null cells is almost completely abrogated [303]. The ejectosome, an F-actin based structure crucial for nonlytic bacterial release, was shown to be absent in *racH*-null cells [304]. Consistently, it was shown that RacH can induce actin polymerization in a cell-free system [301].

The roles of RacH in the actin-based trafficking of vesicles and pathogen motility are reminiscent of the involvement of Cdc42 in the actin-based motility of pathogens in mammalian host cells [305]. Cdc42 has been shown to stimulate formation of the actin comet tail, which is important for intercellular and intracellular motility of various viral and bacterial pathogens [306]. For example, during Vaccinia virus infection, the pathogen activates host Cdc42 via RhoGEF intersectin-1, leading to the stabilization of the N-WASP activity and stimulation of the actin comet tail formation, which enhances the cell-to-cell infection spreading [305]. This Cdc42 activity parallels the role of RacH in the formation of the ejectosome. The main difference is that RacH is essential for the ejectosome formation [304], while actin comet tails can still be formed in the absence of an interaction between Cdc42 and N-WASP, albeit with a lower efficiency: the number of actin tails per cell is reduced [305].

RacD is 54% homologous to human Rac1, lacks the CAAX prenylation motif, and harbours serine-rich insertions close to the C-terminal membrane-association domain. The expression of RacD is present in vegetative cells, rises slightly during early development, and then decreases again [72,272]. Available data show a steady increase of the RacP expression over the first 8 h of development [73,74].

### 4.6. RacE

The most extensively studied *Dictyostelium* Rho GTPase outside of the Rac subfamily is RacE. The *racE* gene was originally isolated in a genetic screen developed to identify genes required for cytokinesis [68]. Activated RacE proved to be essential for cytokinesis in unattached cells [68,287], and it was shown by a pipette aspiration assay that *racE*-null cells have a strongly diminished cortical tension [307]. A molecular mechanism responsible for the RacE involvement in the regulation of cortical integrity was first indicated by the finding that the cortical distribution of actin-binding proteins dynacortin and coronin was altered in *racE*-null cells [308]. More substantial evidence associated RacE with the regulation of 14-3-3 protein, which links the microtubule network to the actin cortex and modulates cortical contractility in cytokinesis via an interaction with the myosin II heavy chain [309,310]. The cortical localization and solubility of 14-3-3 were shown to depend on RacE, whereas the overexpression of 14-3-3 in *racE*-null cells partially rescued their growth, cortical tension, and cytokinesis defects. Although a direct binding between 14-3-3 and RacE was not demonstrated, these data indicated that the RacE/14-3-3 pathway regulates the remodelling and distribution of myosin II bipolar thick filaments and might be partly responsible for the cytokinesis defect of *racE*-null cells [310]. Interestingly, an interaction between RacE and the IQGAP-related protein DGAP1 was also reported, which was independent of the guanine nucleotide bound to RacE [243]. This finding suggests that RacE is instrumental in the regulation of a mechanosensory system that governs myosin II accumulation in the posterior cortex of polarized cells and in the cleavage furrow [311].

As already elaborated in Section 4.1.2, the intramolecular autoinhibition of DRFs is commonly released by the binding of activated Rho-family GTPases [161]. In *Dictyostelium*, it was shown that the mDia1-related DRF ForA contributes to the integrity of the cortical actin layer and localizes to the posterior cortex of polarized interphase cells and to the cleavage furrow of dividing cells [312]. RacE-GTP was identified as the GTPase that binds to and activates ForA, and it was subsequently shown that it also regulates the related cortical DRFs ForE and ForH [313]. Consistently, RacE-GTP localizes to the cell rear and to the cleavage furrow of mitotic cells and is essential for the cortical localization of ForA and ForE. The elimination of all three formins, or of RacE, induced comparable extensive defects in rigidity and architecture of the actin cortex, as demonstrated by aspiration assays and electron microscopy. This resulted in drastic defects in cytokinesis, development, cortical actin flow, and cell polarization, especially under the condition of two-dimensional confinement [313].

Serious defects in the migration and chemotaxis of *racE*-null cells were also identified in an independent line of investigation, focusing initially on the GEF and GAP proteins that regulate RacE [78,314]. It was shown, using a palette of genetic mutations and chemical inhibitors, that RacE and its regulators influence the accuracy of cell orientation in chemotactic gradients and, importantly, that RacE exerts its regulatory role upstream of Ras activation and PIP3 production. How exactly this comes about was revealed when this group uncovered an unexpected mechanism by which the GDP-bound RacE modulates the mTORC2/AKT signalling in GPCR-mediated directed cell migration [315]. The stimulation of cells by a chemoattractant induces the phosphorylation of RacE-GDP at S192 by the GSK3 serine protein kinase. Thereupon, the phosphorylated RacE-GDP assembles with RasC-GTP and mTORC2 into a signalling supercomplex that phosphorylates and thereby activates an important serine/threonine kinase PKB, which in turn phosphorylates multiple substrates that control the dynamics of the actin cytoskeleton and the cell-substratum adhesion [316,317]. In a follow-up study, it was shown that chemoattractant stimulation enables GDP-RacE to oligomerize and to recruit GTP-RasC to assemble RacE-RasC hetero-oligomers that activate mTORC2 [318]. Interestingly, unphosphorylated GTP-RacE inhibits the role of phospho-GDP-RacE in mTORC2 activation by competing with the interaction between phospho-GDP-RacE and GTP-RasC.

**Figure 3:**
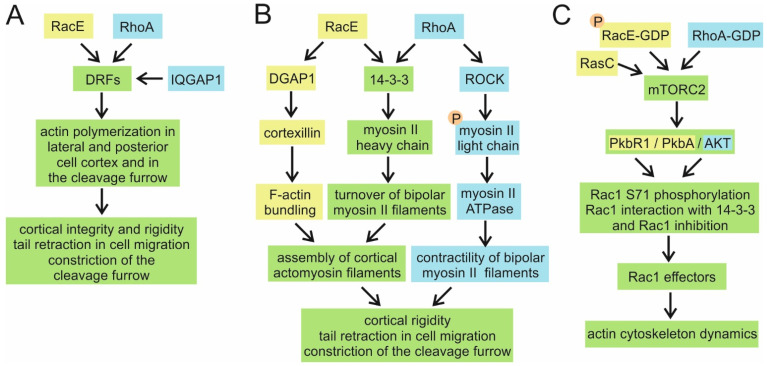
Parallel presentation of major signalling pathways starting from RacE in *Dictyostelium* (*yellow*) and RhoA in mammals (*blue*) and converging onto the actin cytoskeleton regulation. (**A**) Activation of the DRF-mediated actin polymerization. (**B**) Assembly of the actomyosin filaments and activation of their contractility. (**C**) Activation of the mTORC2—AKT signalling pathway. The common elements shared by *Dictyostelium* and mammals are shown in *green*. The default active state of small GTPases is GTP-loaded, except when shown otherwise. Red encircled “P” indicates phosphorylated proteins.

RacE shows considerable sequence similarities with Rho proteins from Metazoa, and it was suggested that it represents an orthologue of mammalian RhoA in *Dictyostelium* (Figure 1 and Figure S2; [35,315]). In particular, the amino acid sequence of the Switch I region, important for the interaction with effectors, which is specific to each type of Rho GTPases, is identical between human RhoA and *Dictyostelium* RacE [50]. Most transduction pathways that convey signals from RacE to the actin cytoskeleton in *Dictyostelium* appear to have their counterparts commencing from RhoA in mammals (Figure 3). RhoA binds to and activates the DRFs mDia1, 2, and 3 in vitro [319,320], and their loss compromised the ultrastructure of the cortical actin cytoskeleton and led to substantial defects in cell polarization and migration [313,321]. Given the alleged interaction between RacE and the IQGAP-related protein DGAP1 in *Dictyostelium*, it is interesting to note that mammalian IQGAP1 is required to target mDia1 to the plasma membrane [254] and enhances the RhoA-mediated activation of mDia1 [322].

Mammalian 14-3-3 family proteins function by binding preferentially to Ser/Thr phosphorylated intracellular proteins, which alters the conformation, activity, and subcellular localization of their binding partners. Until recently, all the data regarding the interaction between Rho GTPases and 14-3-3 proteins were indirect, and it was suggested that 14-3-3 proteins either regulate Rho GTPases through the interaction with RhoGEFs and RhoGAPs or that Rho GTPases control 14-3-3 proteins through PAK1 [323]. However, a direct interaction was recently demonstrated between 14-3-3 and Rac1 that is facilitated by the AKT-mediated phosphorylation of Rac1 at S71 [324]. A similar interaction between 14-3-3 proteins and RhoA has not been shown yet, but it is interesting to note that both RhoA and RacE share the RPLpSYP motif with Rac1, which is very close to the type I consensus motif for the binding of 14-3-3 proteins [325], suggesting that S73 phosphorylation could regulate the interaction of RhoA with 14-3-3 proteins. In *Dictyostelium* and humans alike, 14-3-3 proteins bind directly to the tail of nonmuscle myosin II and inhibit its assembly into bipolar filaments, which increases the soluble fraction of myosin in the cell and promotes myosin turnover [309]. 14-3-3 proteins in mammalian and *Dictyostelium* cells could therefore serve as scaffolds that interact with RhoA and myosin II, thereby promoting the turnover of bipolar myosin II filaments and their reassembly at appropriate intracellular locations. 

Interestingly, an archetypal signalling axis leading from the RhoA-mediated activation of Rho-dependent kinases to the contraction of F-actin-supported bipolar myosin II filaments appears not to be preserved in *Dictyostelium*, although it is apparently preserved in *E. histolytica* [326]. In mammals, the serine/threonine kinase ROCK regulates the phosphorylation of the myosin light chain (MLC) by the direct phosphorylation of MLC and by the inactivation of myosin phosphatase through the phosphorylation of its myosin-binding subunit [327]. Rho kinase and myosin phosphatase thus jointly regulate the MLC phosphorylation, which stimulates cross linking of actin by myosin and enhances actomyosin contractility and stress fibre formation in nonmuscle cells [328]. In *Dictyostelium*, myosin motor activity is regulated by the phosphorylation of the regulatory light chain through MLCK A [329]. Unlike conventional light chain kinases, this enzyme is not regulated by calcium but is activated by cGMP-induced phosphorylation via an upstream kinase and subsequent autophosphorylation. No Rho-dependent kinases involved in the regulation of the actomyosin contraction were identified in *Dictyostelium*. On the other hand, the unusual involvement of RacE-GDP in the regulation of the mTORC2-AKT pathway does appear to have its equivalent in mammalian cells, although additional work is needed to elucidate this pathway involving RhoA-GDP in more detail [315].

In summary, although RacE and RhoA probably diverged independently from the core Rac group in LECA, they participate in remarkably conserved signalling pathways in Amoebozoa and animals (Figure 3). One of these pathways activates actin polymerases from the DRF group (Figure 3A), whereas the other regulates the contractile machinery propelled by myosin II (Figure 3B). Both pathways are essential for the regulation of cell polarization, since the constitutive proteins localize in the lateral and the posterior nonprotrusive cell cortex and determine its ultrastructure, rigidity, and contractility. It appears, therefore, that in the two systems, RhoA and its functional counterpart RacE are involved in setting up an appropriate organization of the actin–myosin contractile filamentous structures, e.g., the retractile tail of migrating cells, the contractile cytokinetic ring, and the stress fibres anchored in focal adhesions. Consequently, the inhibition of RhoA leads to severe defects in the cortical rigidity, cell migration, and cell division in mammalian cells [330,331,332], resembling the aforementioned consequences of the *racE* knockout in *Dictyostelium*. Importantly, RacE/RhoA participate in the posterior/central pathways in their GTP-bound form and regulate effectors closely associated with the actin cytoskeleton (RacE—DRFs—actin, RacE—14-3-3—myosin II/actin). On the other hand, they also participate in an anterior pathway close to the protruding cell front in their GDP-bound form and transduce the signal by oligomerizing with Ras much more upstream from actin (RacE/RasC—mTORC2—AKT—intermediates—actin cytoskeleton) (Figure 3C). Therefore, the two modes of RacE/RhoA action are separated spatially, temporally, and biochemically from each other. This dual role of RacE/RhoA is therefore somewhat reminiscent of the dual role played by Rac1 GTPases in the front and the back of polarized *Dictyostelium* cells [122,333].

### 4.7. Other Rho GTPases (G, L, I, J, M, N, O, and Q)

There is very little information available about the remaining eight Rho GTPases from *D. discoideum*, a group somewhat related to mammalian Miro and BTB GTPases (RacI, J, M, N, O, and Q), and an unrelated group of mutually similar RacG and RacL (Figure 1). 

RacG is constitutively expressed in growing and developing cells, with a decrease in expression after the slug stage [272,300]. Its expression is also induced during sexual maturation followed by a reduction immediately after the gamete fusion, suggesting that it may have a specific role in interactions between sexually competent cells [75]. RacG is localized uniformly in the cell cortex, and it is also found in long, highly motile filopodia induced by its overexpression [334]. It is also enriched at the rim of the nascent phagocytic cups, and its overexpression enhances phagocytosis efficiency [334]. However, *racG*-null cells did not show any defects in growth, large-scale endocytosis, cytokinesis, or development. The only observed defect was an impaired migration of mutant cells in the cAMP gradient, suggesting that RacG likely shares its roles in the regulation of cell morphology, phagocytosis, and chemotaxis with other Rac proteins [334].

RacI, RacJ, and RacL are expressed throughout the life cycle, and while the expression of RacI culminates in early development, RacJ and RacL reach their maximum expression in the late stages of development [72,73,74,272]. It has therefore been suggested that RacJ and RacL, together with RacG, may have a role in the regulation of the multicellular development of *D. discoideum* [75]. Remarkably, RacJ has a divergent switch I region [272]. The expression levels of RacM and RacQ are highest at the first hour of development, whereas RacN and RacO have the highest expression at the fifth hour of development [73,74].

## 5. Concluding Remarks

The actin cytoskeleton is an ancient constituent of living cells and epitomizes the fundamental principle of the noncovalent “polymerization” of globular protein units into “cytomotive” filamentous structures, already present in prokaryotes [335,336,337,338]. The recent discovery of a protoactin along with a subset of proteins involved in actin polymerization and depolymerization in Asgard archaea established that a dynamic actin cytoskeleton predates the advent of eukaryotes [339,340,341,342,343]. This and other related findings [144,344] strongly support the view that the basic constitutive and regulatory elements of the actin cytoskeleton, the principles of its organization and polarity, as well as the actin-dependent phenotypes, were already established in the common ancestor of all eukaryotes [343,345]. Starting from there, eukaryotic clades have infused a vast variety of ecological niches and adopted a wide repertoire of lifestyles, adjusting the composition and functionality of their actin cytoskeleton accordingly [346]. Among them, remarkably similar traits reliant on the actin cytoskeleton have evolved in some representatives of Amoebozoa and in several classes of animals, in particular mammalian cells, and primarily motile cells of the immune system [347,348]. It appears, therefore, that the functional modules that shape the actin cytoskeleton, encompassing actin-binding proteins and upstream signalling proteins, coevolved in a similar manner in Amoebozoa and Metazoa and, although differing in details, share fundamental structural and operational principles.

A large number of small GTPases, including putative representatives of the Rho family, were identified in Lokiarchea [349,350]. These RhoLs, however, lack some typical signatures of eukaryotic Rhos, e.g., the CAAX motif essential for prenylation and binding to the membrane, and no obvious orthologues of eukaryotic GEFs and GAPs have been detected in archaeal genomes so far [350,351]. Since Rho GTPases are also present in all major extant eukaryotic clades, there is little doubt that they were also present in LECA [4]. In Metazoa, the number of Rho GTPases and the cellular processes in which they participate grew with the increase of complexity and the number of cell types in multicellular animals, leading to the present repertoire of Rho GTPases in mammals [4,34]. Somewhat surprisingly, our analysis indicates that a similar, but independent, diversification took place in dominantly unicellular Amoebozoa (Figure 1). Although they evolved independently, many of these proteins perform similar tasks and are involved in the regulation of similar signalling pathways as in animals. Whereas many of the cytoskeletal functional modules were already present in the last common ancestor of Amoebozoa and Metazoa, the functional equivalence of at least some independently evolved GTPases, e.g., of RhoA in human and RacE in *Dictyostelium*, is remarkable (Figure 3).

We analysed the phylogeny of Rho GTPases from 12 amoebozoan species belonging to four classes: Eumycetozoa (seven species, e.g., *Dictyostelium discoideum*, social amoeba undergoing aggregation into a transient multicellular form; [352,353]), Archamoeba (three species, e.g., *Entamoeba histolytica*, human pathogen and an obligate amitochondriate anaerobe; [354]), Centramoebia (*Acanthamoeba castellanii*, an ubiquitously distributed solitary amoeba and an opportunistic parasite; [355]), and Variosea (*Planoprotostelium aurantium* var. *fungivorum*, unicellular amoebozoon that sporulates by making an unicellular sporocarp; [356]). The number of Rho GTPases encoded in the genomes of typical representatives of the examined amoebozoan classes correlates roughly with their lifestyles. *D. discoideum*, as a free-living aerobic amoeba whose life cycle encompasses unicellular and multicellular stages, several modes of migration, and large-scale endocytosis, cell differentiation, and sporulation [357], and *E. histolytica*, which interacts with the complex environment of the host organisms and features a rich repertoire of actin-based structures [358], encode 20 and 19 Rho GTPase proteins, respectively, comparable to 20 Rho GTPases in humans. On the other hand, the free-living, solitary *A. castellanii* and *P. aurantium* encode only five and six Rho GTPases, respectively, comparable to five in the sponge *Amphimedon queenslandica* (Porifera) and seven in the sea anemone *Nematostella vectensis* (Cnidaria). It should be emphasized that the divergence of the surveyed amoeba classes is ancient, and even the branching of *D. discoideum* and *E. histolytica*, which are both classified into the Conosa subphylum, has been estimated to be greater than between animals and fungi [65]. It appears, therefore, that a correlation between the complexity of cellular processes involving the actin cytoskeleton and richness of the Rho GTPase repertoire has general validity across eukaryotes. In accord with this notion, the genome of the free-living amoeboflagellate *Naegleria gruberi*, which belongs to a varied and ubiquitous protist clade of Heterolobosea that diverged from other eukaryotic lineages over a billion years ago, encodes at least 23 and possibly more Rho GTPases [33,359]. On the other hand, the mammalian intestinal parasite from another deep-branching eukaryotic lineage, *Giardia lamblia*, contains a highly reduced actin cytoskeleton, lacking canonical actin-binding proteins, and a single Rho GTPase, glRac [360]. Intriguingly, glRac appears to exert effects on the *Giardia* actin cytoskeleton via a 14-3-3 orthologue [361]. Consistent with the expansion of Rho family GTPases in Amoebozoa and Heterolobosea, the species belonging to these and other clades that share the amoeboid phenotype also encode an excess of Dbl-RhoGEFs [362]. It has been shown that this expansion appeared independently in Amoebozoa, Heterolobosea, Parabasalia, and Rhizaria, indicating a convergent evolution of Dbl-like RhoGEFs, probably driven by diversity of extracellular stimuli that these amoeboid protists were exposed to. The authors conclude that Dbl-like RhoGEFs are functionally involved in the acquisition of the amoeboid phenotype [362].

Finally, we would like to comment on an enduring perception that *D. discoideum* contains only members of one out of the three archetypical small Rho GTPase classes, the Rac group [35], although suggestions have been made, mainly on the basis of functional data alone, that RacE and RacC represent bona fide orthologues of Rho and Cdc42, respectively [69,82,313,315,363,364]. As elaborated in Section 3, our phylogenetic analysis does not sufficiently support a homology even between the *Dictyostelium* and mammalian Rac proteins, and the dilemma between their common ancestry or convergent evolution remains unsolved. The numerous parallelisms between the signalling pathways that involve the corresponding GTPase classes in these organisms, however, suggest that they represent functional equivalents or counterparts, regardless of their evolutionary relationships. Bona fide functional equivalents of Rac1, RhoA, and Cdc42 are also present in *E. histolytica* and *P. aurantium*, whereas *A. castellanii* apparently lacks a typical Rho s.s. While the Cdc42, Rho, and BTB groups underwent considerable expansion in mammals, the most remarkable feature in *D. discoideum*, other eumycetozoans, and *E. histolytica* is the appearance of a group of closely related RacH, D, and P, which have no obvious mammalian equivalents. Despite the differences between individual taxa, the combined evidence from the sequenced and analysed genomes of basal Metazoa [34], Amoebozoa (this work), other early branching eukaryotes [359], and Asgard archaea [349,350] suggests that the archetypical Rho family GTPases were already present in LECA, together with other basic structural and regulatory elements of the actin cytoskeleton [343,346,365].

## Supplementary Materials

The following are available online at https://www.mdpi.com/article/10.3390/cells10071592/s1, Supplementary File 1: Figures S1–S3: Multiple sequence alignments of Rho GTPases, Table S1: Rho GTPases in Amoebozoa; Supplementary File 2: Accession numbers and amino acid sequences of Rho proteins from selected species.

## Abbreviations

ABD	actin-binding domain
ABI	ABL interactor
Abp1	actin-binding protein 1
ACA	adenylyl cyclase A
AID	autoinhibitory domain
Arp2/3	actin related protein 2/3
BTB	broad-complex, tamtrack, bric à brac
CCP	clathrin-coated pit
CCV	clathrin-coated vesicle
CHD	calponin homology domain
CI	cortexillin I
CII	cortexillin II
CLIP-170	cytoplasmic linker protein-170
CME	clathrin-mediated endocytosis
CRIB	Cdc42/Rac interactive binding
CYRI	CYFIP-related Rac interactor
DAD	Diaphanous-autoregulatory domain
DD	dimerization domain
DID	Diaphanous-inhibitory domain
DRF	Diaphanous-related formin
DUF1394	domain of unknown function
EGFR	epidermal growth factor receptor
ERK	extracellular signal-regulated kinase
FcγR	Fcγ receptor
FH1	formin homology 1
FH2	formin homology 2
FH3	formin homology 3
FRET	fluorescence resonance energy transfer
GAP	GTPase activating protein
GEF	guanine–nucleotide exchange factor
GBD	GTPase binding domain
GPCR	G protein-coupled receptor
GRD	GAP-related domain
HSPC300	hematopoietic stem/progenitor cell protein 300
JMY	junction-mediating and -regulatory protein
MEF	mouse embryonic fibroblast
MHCK	myosin II heavy chain kinase
MIHCK	myosin I heavy chain kinase
MLC	myosin II light chain
MLCK	MLC kinase
NAP1	Nck-associated protein 1
NPF	nucleation promoting factor
N-WASP	neural WASP
PAK	p21-activated kinase
PBD	p21 binding domain
PH	pleckstrin homology
PI(3,4)P2	phosphatidylinositol (3,4)-bisphosphate
PI3K	phosphatidylinositol 3-kinase
PIP3	phosphatidylinositol (3,4,5)-trisphosphate
PIR121	121F-specific p53 inducible RNA
PKB	protein kinase B
PRD	proline rich domain
REM	Rho effector homology
RGCT	RasGAP C-terminus
RhoGDI	Rho GDP-dissociation inhibitor
Rif	Rho in filopodia
RKH	ROK-kinectin homology
SRA1	specifically RAC1-associated protein 1
VEGF	vascular endothelial growth factor
VEGFR2	VEGF receptor 2
WASH	WASP and SCAR homologue
WASP	Wiskott–Aldrich syndrome protein
WAVE/SCAR	WASP family verprolin-homologous protein/suppressor of cAMP receptor
WCA	WASP-homology-2, also known as verprolin-homology, cofilin-homology, or connecting or central, acidic
WHAMM	WASP homolog associated with actin, membranes, and microtubules
WRC	WAVE regulatory complex
Y2H	yeast-two-hybrid
