# Supplementary material for: Regulation of the Actin Cytoskeleton via Rho GTPase Signalling in Dictyostelium and Mammalian Cells: A Parallel Slalom
Source: Cells. 2021 Jun 24;10(7):1592. doi: 10.3390/cells10071592 (PMC8305917; doi:10.3390/cells10071592)
Supplement: Supplementary file 1 [file cells-10-01592-s001.zip › cells-1258406-Supplementary File/cells-1258406-Supplementary File 1.pdf]

# SUPPLEMENTARY MATERIALS

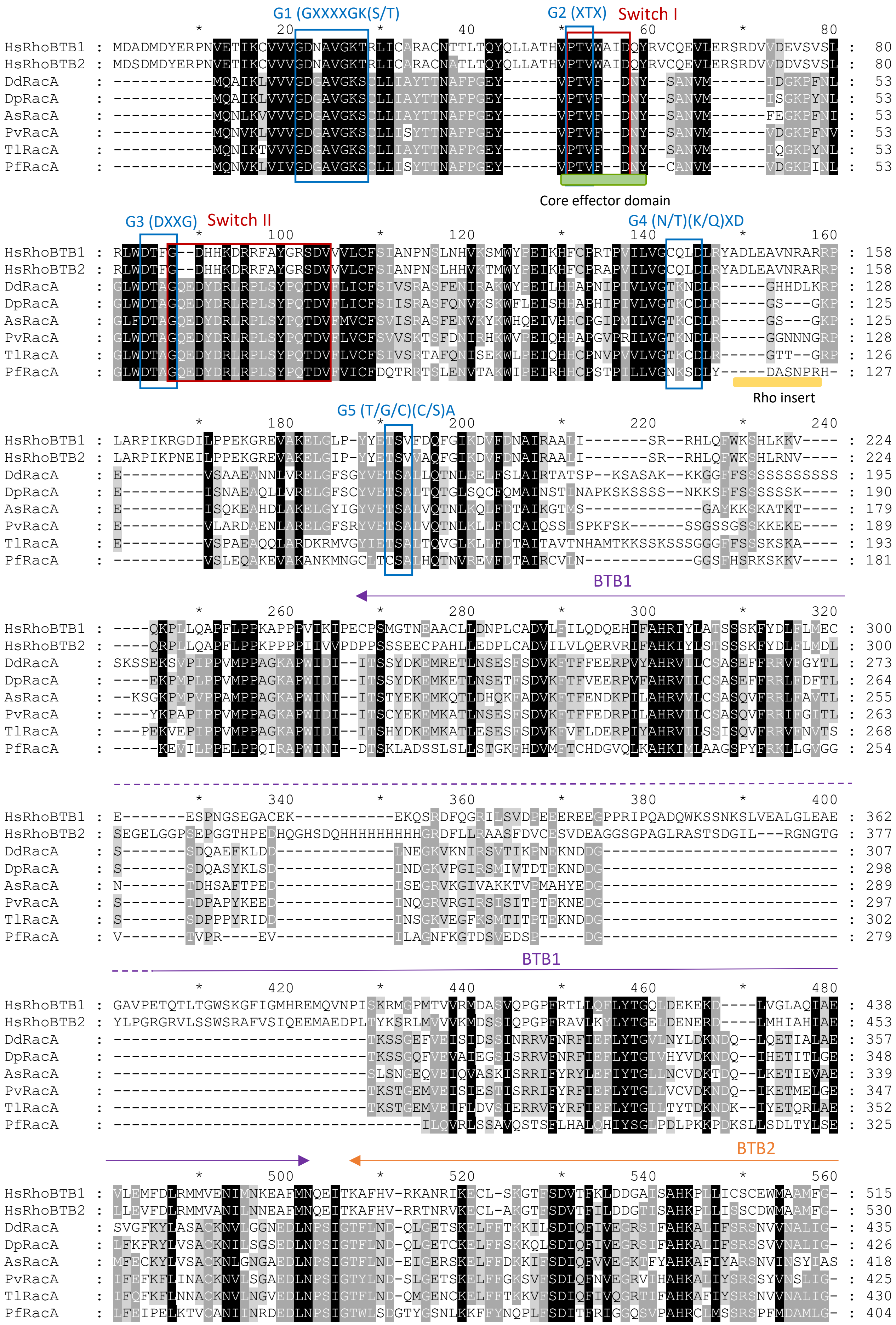

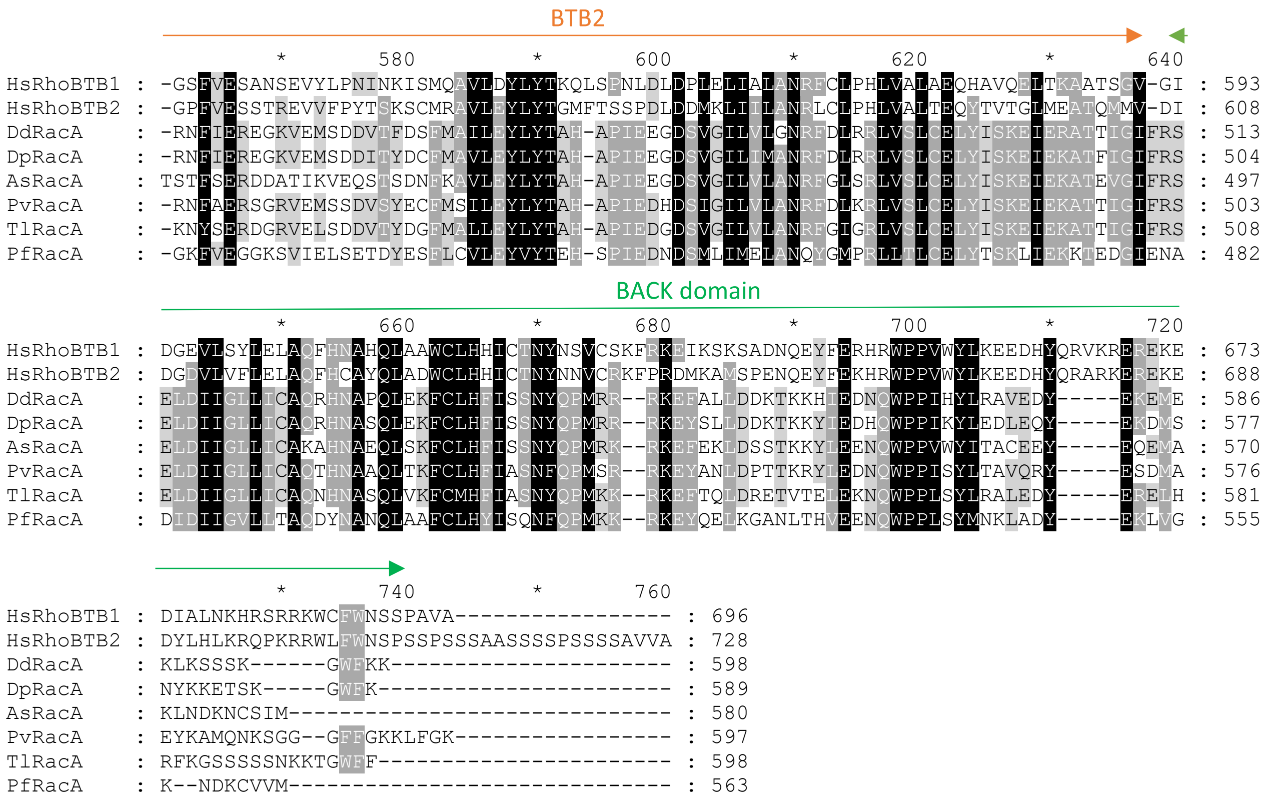


Figure S1. Sequence alignment of RhoBTB 1 and 2 and RacA subfamily proteins. The amino acid sequences were aligned using the MUSCLE algorithm [62]. Multiple alignments were visualized and edited using the GeneDoc software 2.7. The amino acid residues were shaded using the conserved residue shading mode set to level 4 with default settings. Similar residues conserved in all proteins have a dark shading. The residues with 100%, 80%, and 60% conservation are shaded as white letters on the black background, white letters on the dark-grey background, and black letters on the light-grey background, respectively. Specific regions are indicated as follows: the G boxes are framed in blue (G1, G2, G3, G4, and G5 with the respective consensus motifs), the switch I and switch II regions are framed in red, a purple line designates the first BTB domain, an orange line the second BTB domain, and a green line the C-terminus back domain. The core effector domain and the Rho insert are indicated by green and orange bars, respectively.

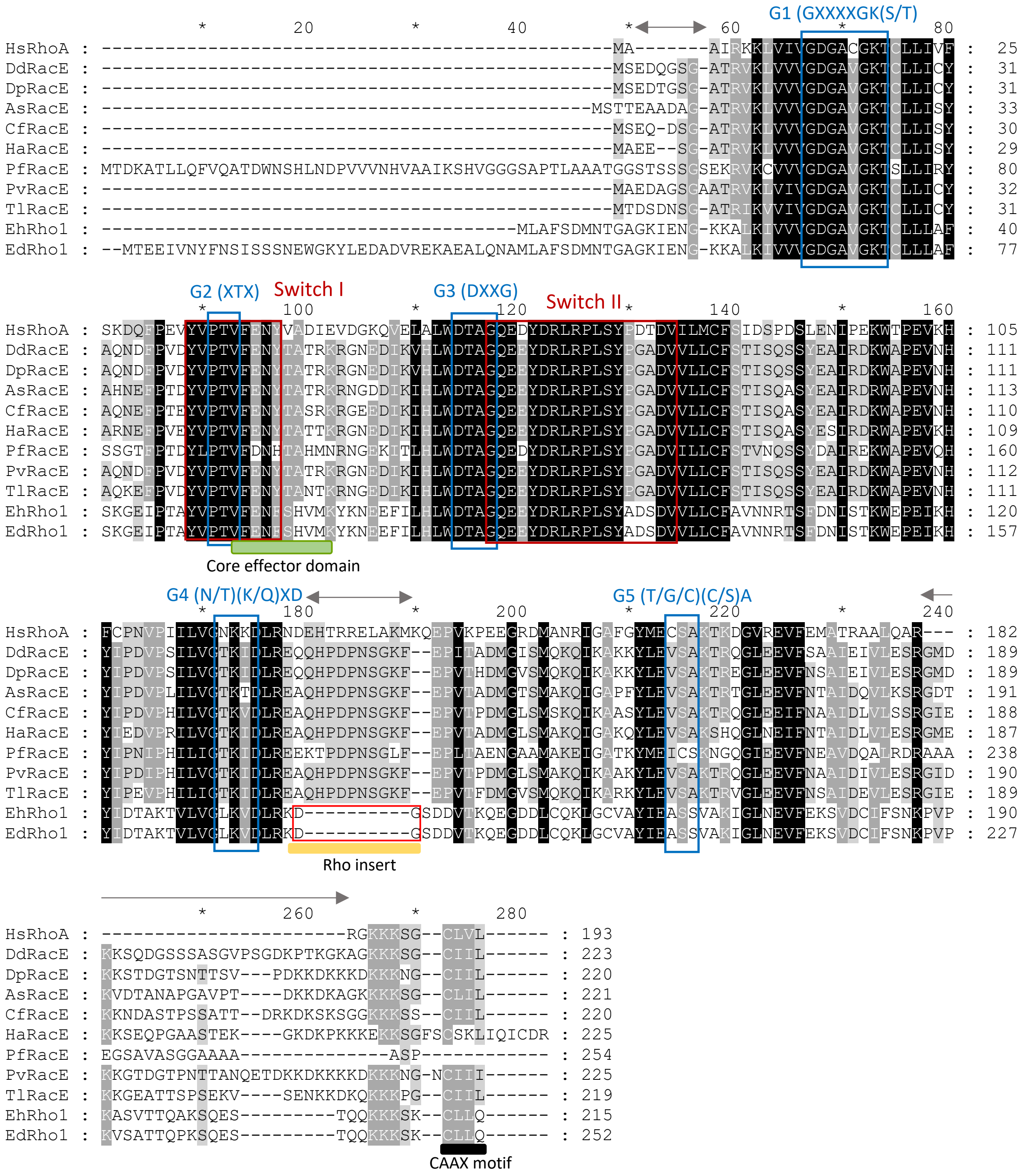


Figure S2. Sequence alignment of RhoA and RacE subfamily proteins. The amino acid sequences were aligned using the MUSCLE algorithm [62]. Conserved sequences are shaded using the GeneDoc software 2.7 (see Figure S1). Specific regions are indicated as follows: the G boxes are framed in blue (G1, G2, G3, G4, and G5 with the respective consensus motifs), and the switch I and switch II regions are framed in red. The core effector domain, the Rho insert, and the conserved CAAX motif at the C-terminus are indicated by green, orange and black bars, respectively. The grey lines at the N- and C-terminus represent the important deletions in human or entamoeban proteins.

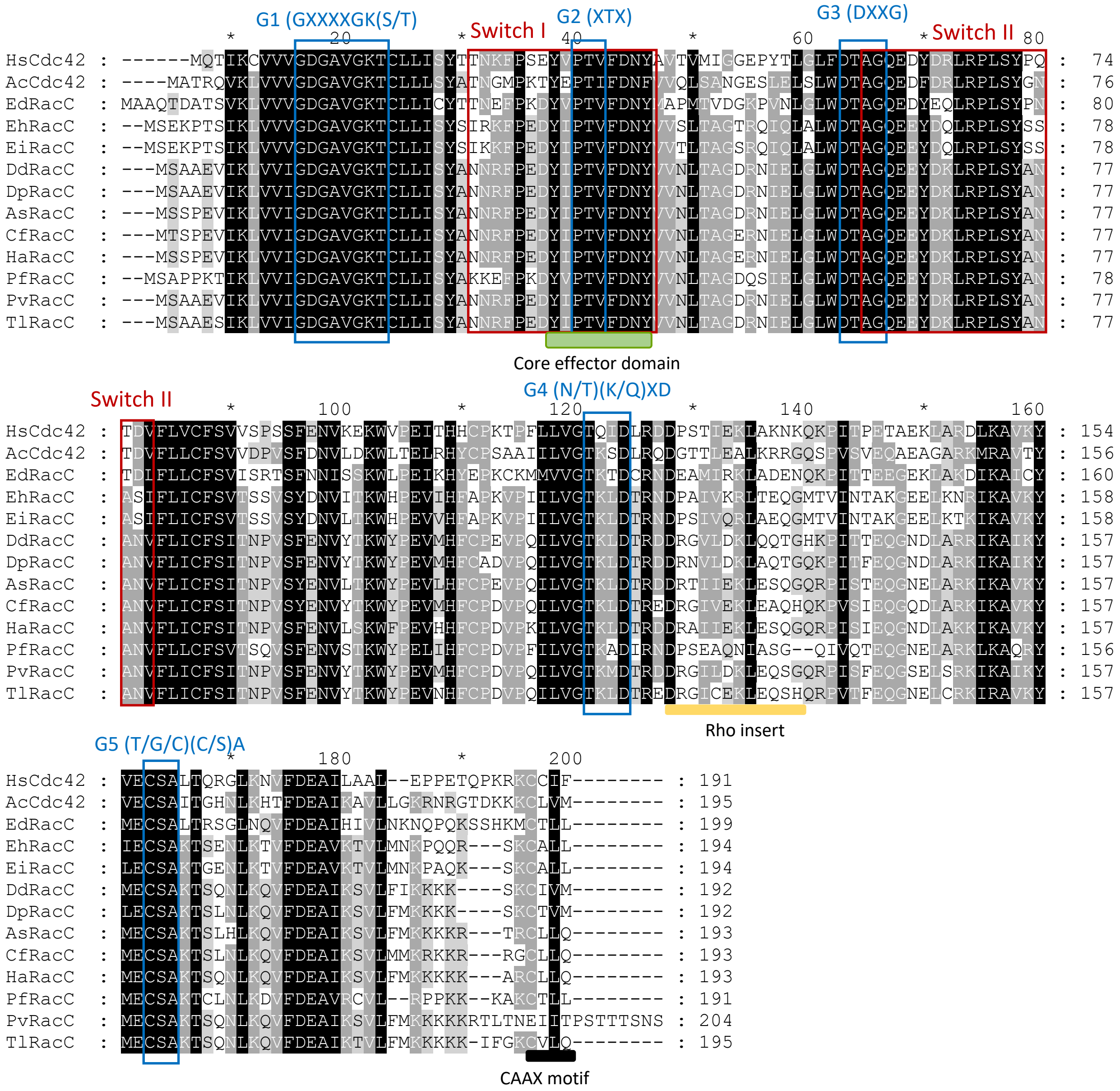


Figure S3. Sequence alignment of Cdc42 and RacC subfamily proteins. The amino acid sequences were aligned using the MUSCLE algorithm [62]. Conserved sequences are shaded using the GeneDoc software 2.7 (see Figure S1). The amino acid residues are shaded as in Figure S1. Specific regions are indicated as follows: the G boxes are framed in blue (G1, G2, G3, G4, and G5 with the respective consensus motifs), and the switch I and switch II regions are framed in red. The core effector domain, the Rho insert, and the conserved CAAX motif at the C-terminus are indicated by green, orange and black bars, respectively.

Table S1. Rho GTPases in Amoebozoa.

| Amoebozoa | | | | | | |
|---|---|---|---|---|---|---|
| | **Phylum** | **Organism** | **Rho GTPases** | | | |
| Conosa | **Archamoebea** | *Entamoeba histolytica* | EhCdc42 | EhRacG | XP_653308.1 | XP_652457.1 |
| | | | EhRacA | EhRacH | XP_649502.1 | XP_648766.1 |
| | | | EhRacB | EhRho1 | XP_656301.1 | XP_654700.1 |
| | | | EhRacC | XP_653885.1 | XP_656258.1 | XP_656370.1 |
| | | | EhRacD | XP_653812.1 | XP_652660.1 | |
| | | *Entamoeba dispar* | EdRacA-like | EdRacG-like | XP_001739099.1 | XP_001738433.1 |
| | | | EdRacB-like | EdRacH-like | XP_001737112.1 | XP_001734489.1 |
| | | | EdRacC-like | EdRho1-like | XP_001737038.1 | XP_001739566.1 |
| | | | EdRacD-like | XP_001736788.1 | XP_001736684.1 | XP_001741992.1 |
| | | *Entamoeba invadens* | EiCdc42-like | EiRacD-like | XP_004185779.1 | XP_004258224.1 |
| | | | EiRacA-like | EiRacG-like | XP_004258338.1 | XP_004185920.1 |
| | | | EiRacB-like | EiRacH-like | XP_004183588.1 | XP_004184178.1 |
| | | | EiRacC-like | XP_004261490.1 | XP_004261195.1 | XP_004254590.1 |
| | **Dictyostelia** | *Dictyostelium discoideum* | DdRac1A | DdRacF1 | DdRacG | DdRacM |
| | | | DdRac1B | DdRacF2 | DdRacH | DdRacN |
| | | | DdRac1C | DdRacC | DdRacI | DdRacO |
| | | | DdRacA | DdRacD | DdRacJ | DdRacP |
| | | | DdRacB | DdRacE | DdRacL | DdRacQ |
| | | *Dictyostelium purpureum* | DpRac1A-like | DpRacD-like | DpRacL-like | DpRacU-like |
| | | | DpRac1B-like | DpRacE-like | DpRacP-like | DpRacV-like |
| | | | DpRacB-like | DpRacG-like | DpRacR-like | DpRacW-like |
| | | | DpRacA-like | DpRacH-like | DpRacS-like | |
| | | | DpRacC-like | DpRacJ-like | DpRacT-like | |
| | | *Acytostelium subglobosum* | AsRac1A-like | AsRacC-like | AsRacH-like | |
| | | | AsRac1B-like | AsRacD-like | AsRacL-like | |
| | | | AsRacA-like | AsRacE-like | AsRacP-like | |
| | | | AsRacB-like | AsRacG-like | | |
| | | *Cavenderia fasciculata* | CfRac1A-like | CfRacC-like | CfRacH-like | |
| | | | CfRac1B-like | CfRacD-like | CfRacL-like | |
| | | | CfRacB-like | CfRacE-like | CfRacP-like | |
| | | *Heterostelium album* | HaRac1B-like | HaRacC-like | HaRacH-like | |
| | | | HaRac1C-like | HaRacD-like | HaRacL-like | |
| | | | HaRacB-like | HaRacE-like | HaRacP-like | |
| | | | HaRacF2-like | HaRacG-like | | |
| | | *Polysphondylium violaceum* | PvRac1A-like | PvRacC-like | PvRacL-like | |
| | | | PvRac1B-like | PvRacD-like | PvRacP-like | |
| | | | PvRacA-like | PvRacE-like | | |
| | | | PvRacB-like | PvRacH-like | | |
| | | *Tieghemostelium lacteum* | TlRac1A-like | TlRacD-like | TlRacL-like | |
| | | | TlRacA-like | TlRacE-like | TlRacP-like | |
| | | | TlRacB-like | TlRacG-like | | |
| | | | TlRacC-like | TlRacH-like | | |
| | **Variosea** | *Planoprotostelium fungivorum* | PfRac1-like | PfRacA-like | PfRacE-like | |
| | | | PfRac1B-like | PfRacC-like | PfRacH-like | |
| Lobosa | **Discosea** | *Acanthamoeba castellanii* | AcCdc42-like | AcRac1B-like | AcRac3-like | |
| | | | AcRac1 | AcRac2-like | | |

Rho GTPase genes from *Dictyostelium discoideum* and *Entamoeba histolytica* were identified based on publicly available information and additional analysis at the National Center for Biotechnology Information database (NCBI). Amino acid sets of Rho GTPases from both organisms were used as queries for a sequence similarity search in the NCBI database with the *blastp* algorithm (https://blast.ncbi.nlm.nih.gov/Blast.cgi). Predicted protein homologs from other Amoebozoa with fully annotated genomes that showed the highest percent identity are listed. Proteins previously reported in the literature were named according to already published data. Names of unpublished but predicted

proteins from other Amoebozoa were chosen based on homology and designated by adding ”-like” to their names. Accession numbers of all sequences reported in the table can be seen in the Supplementary File 2. (Accession numbers and amino acid sequences of selected species).
